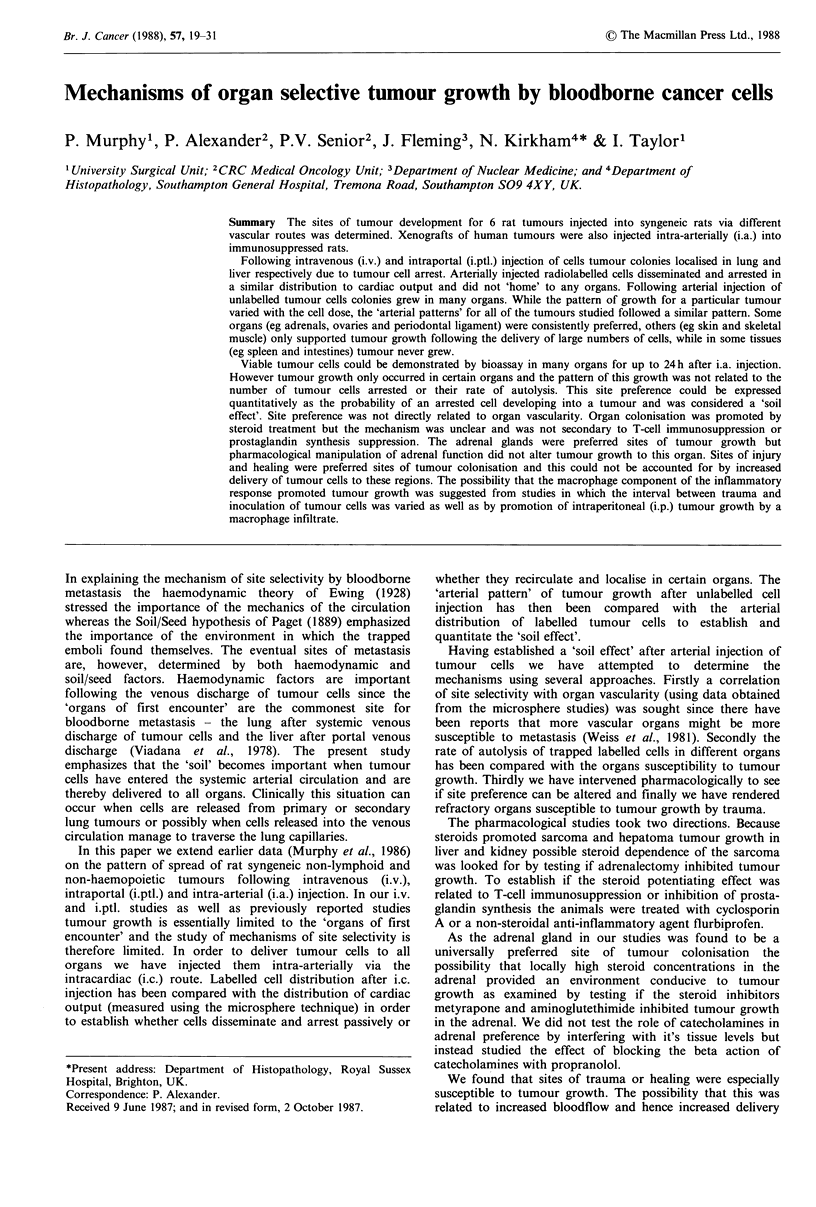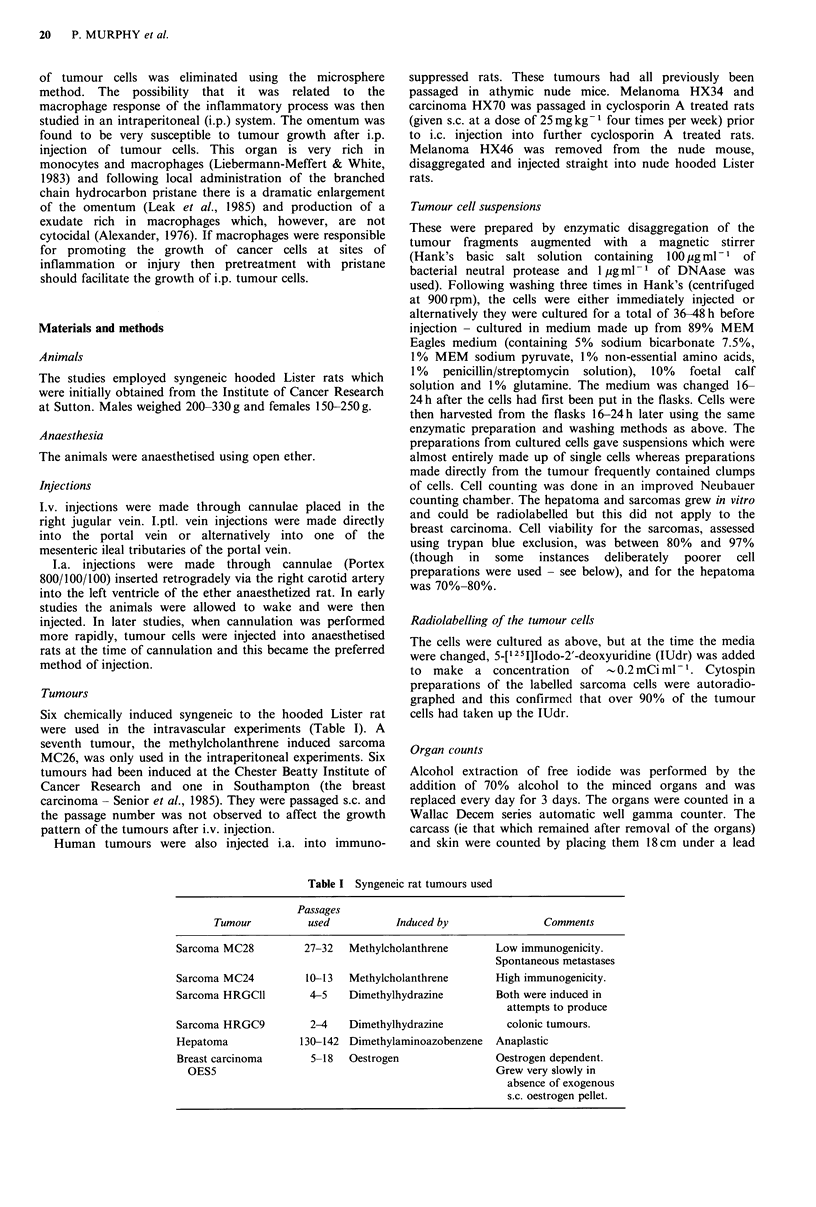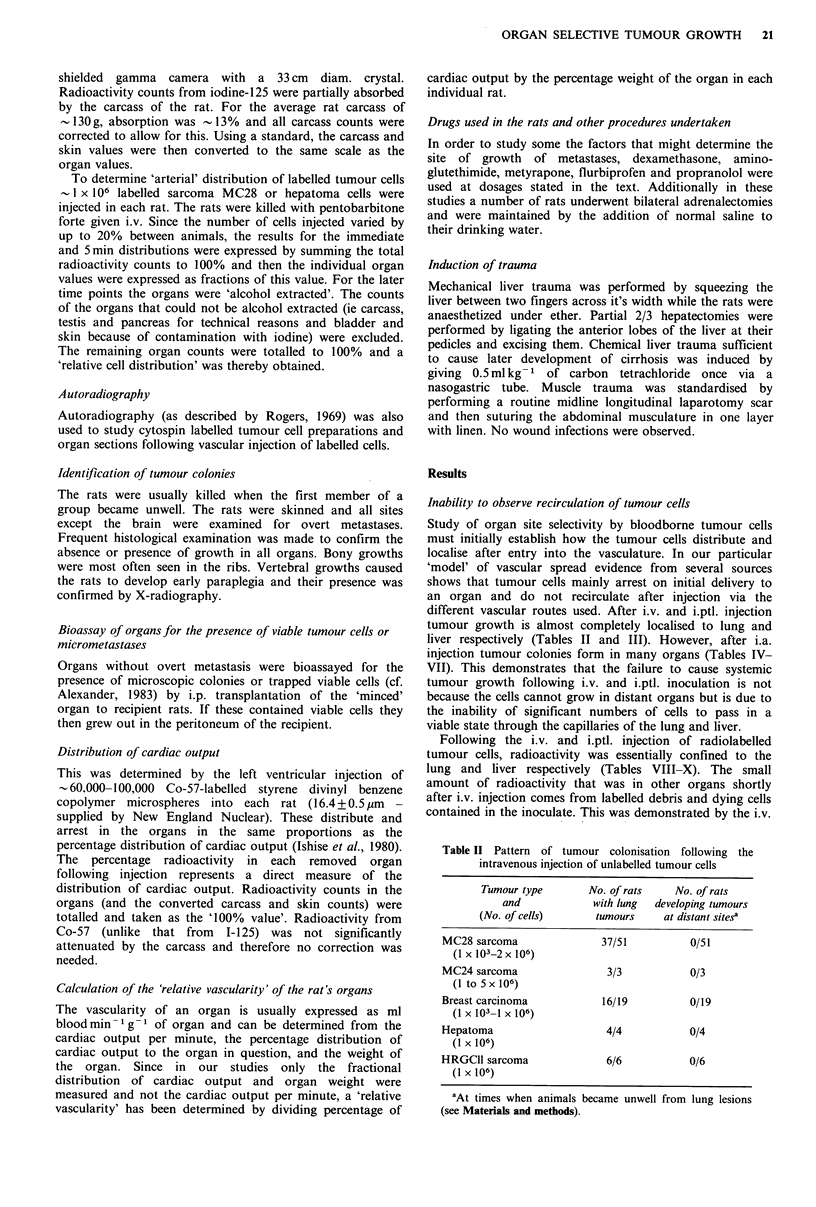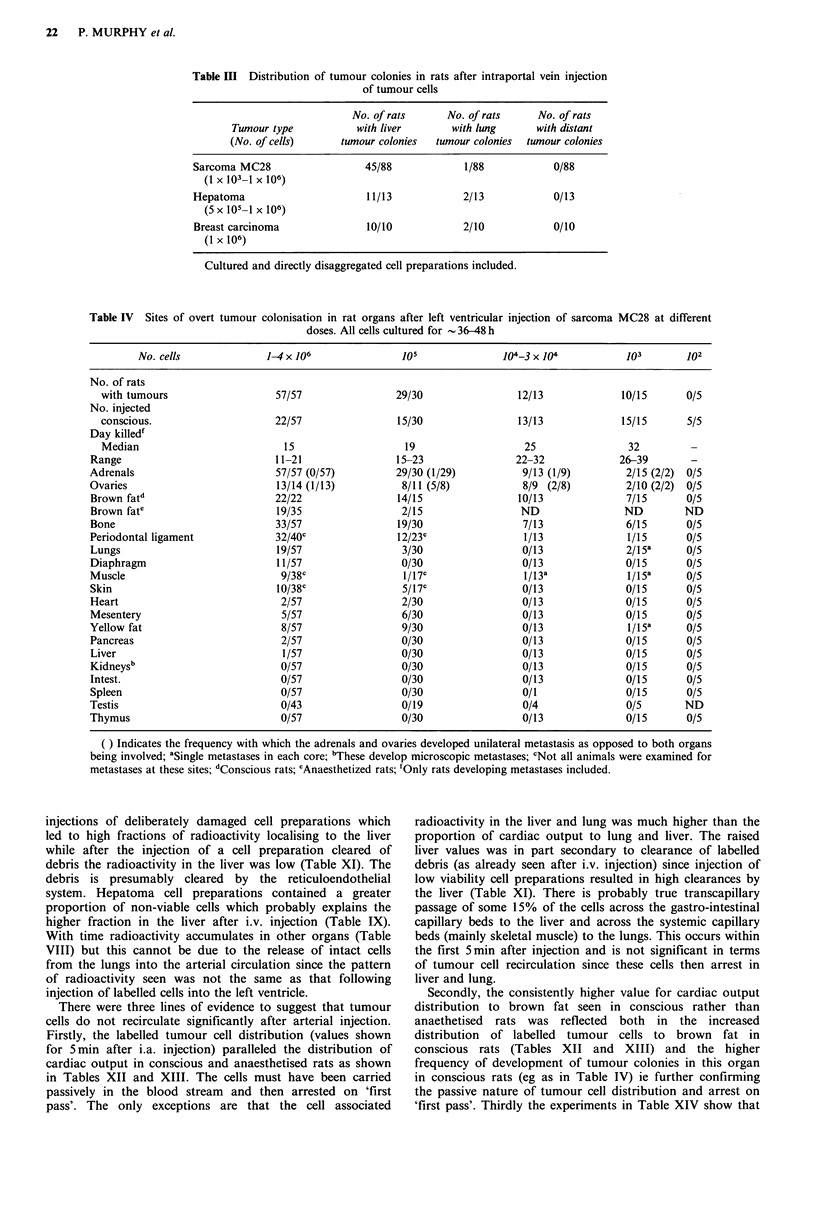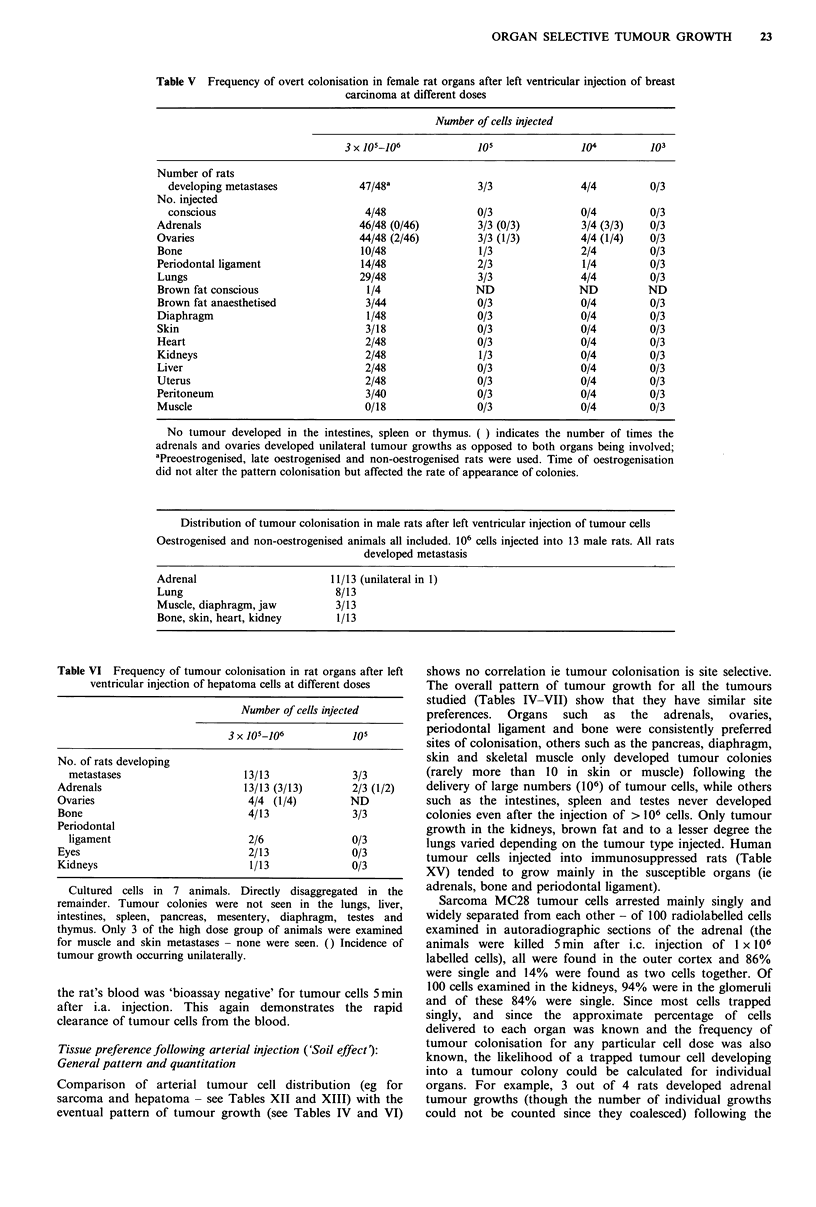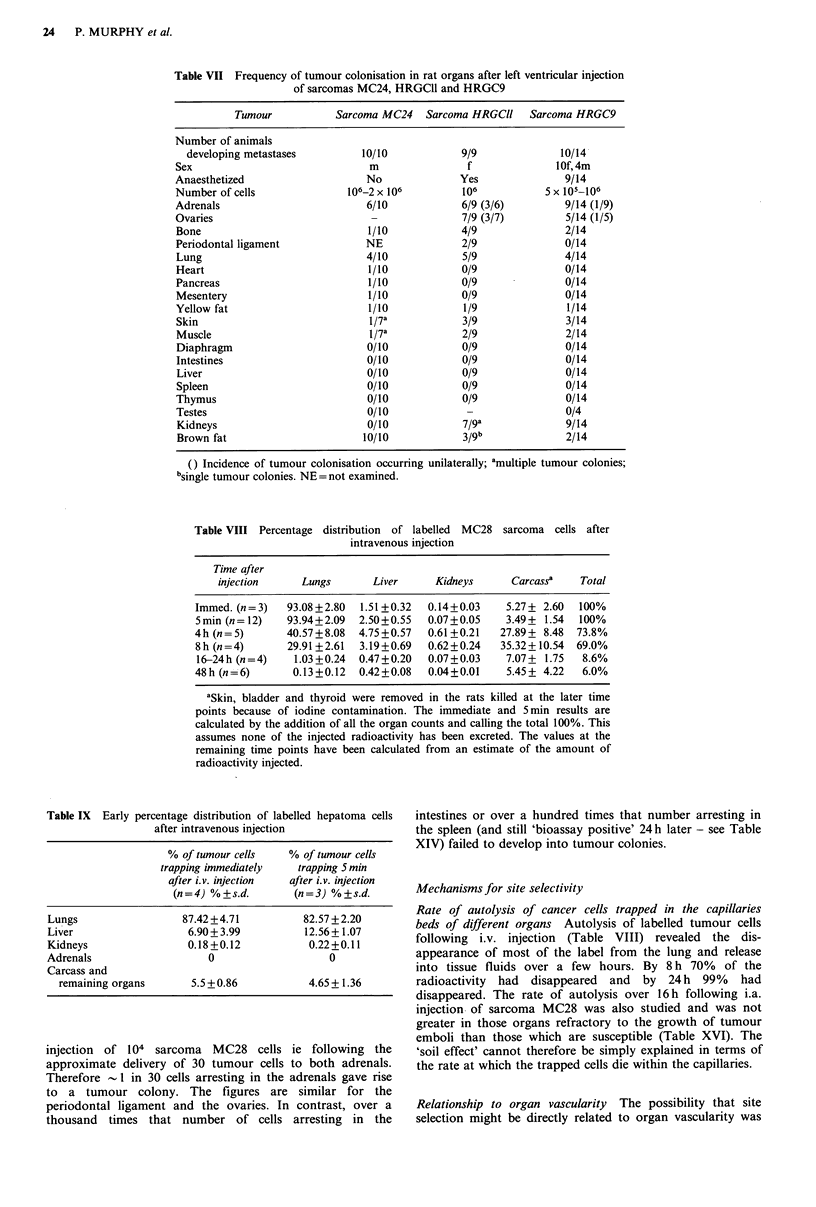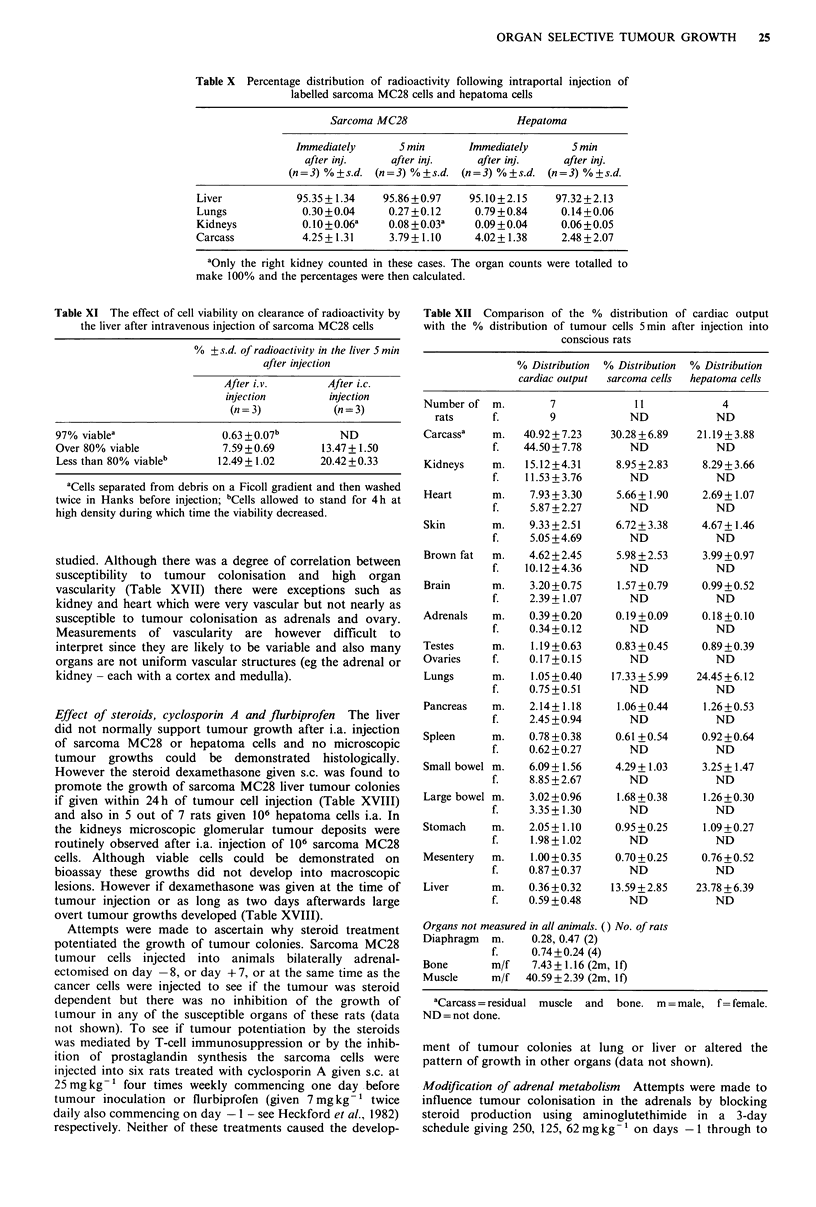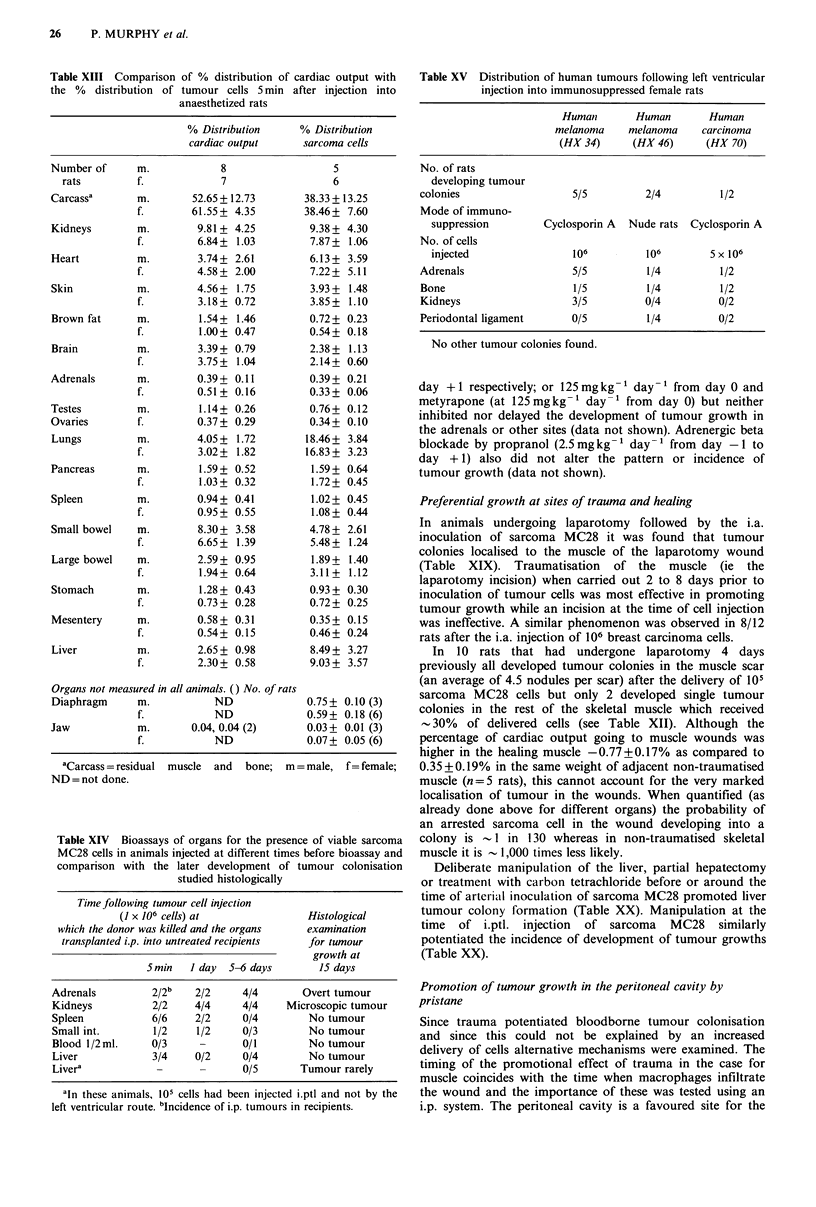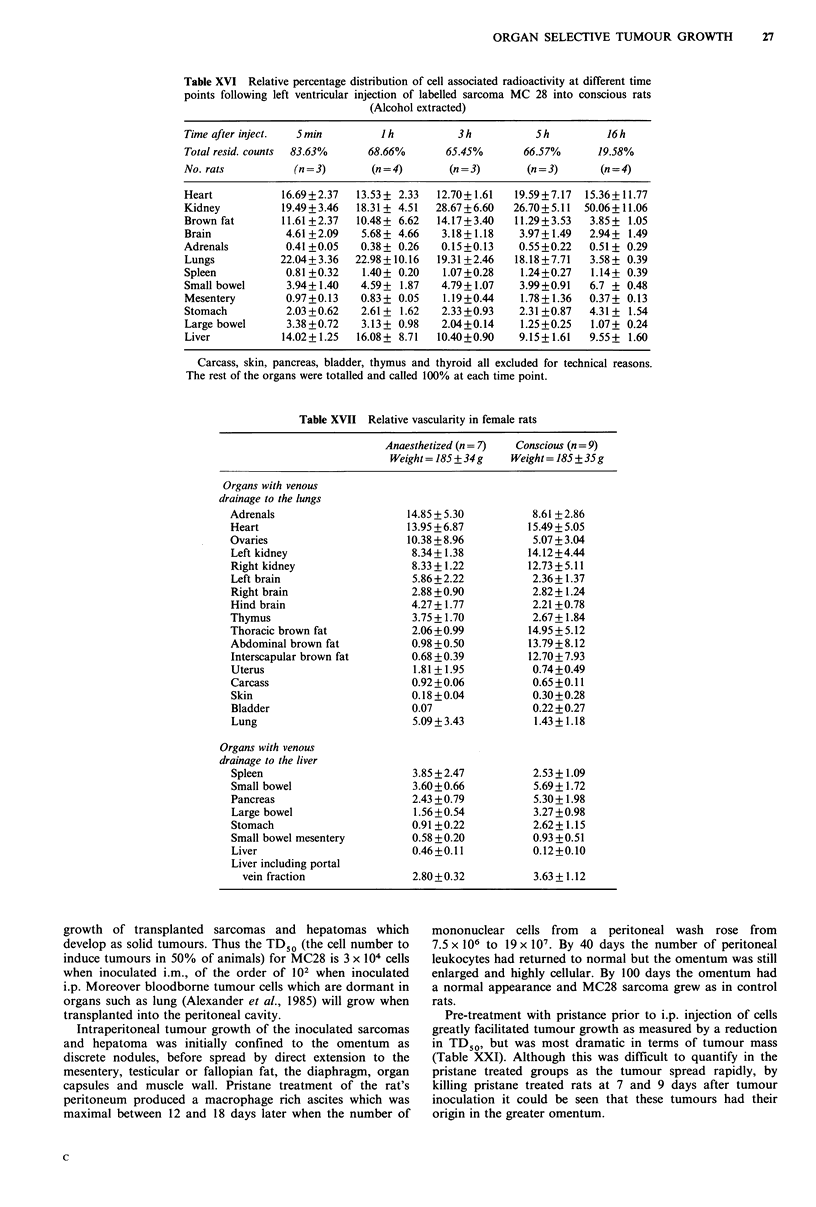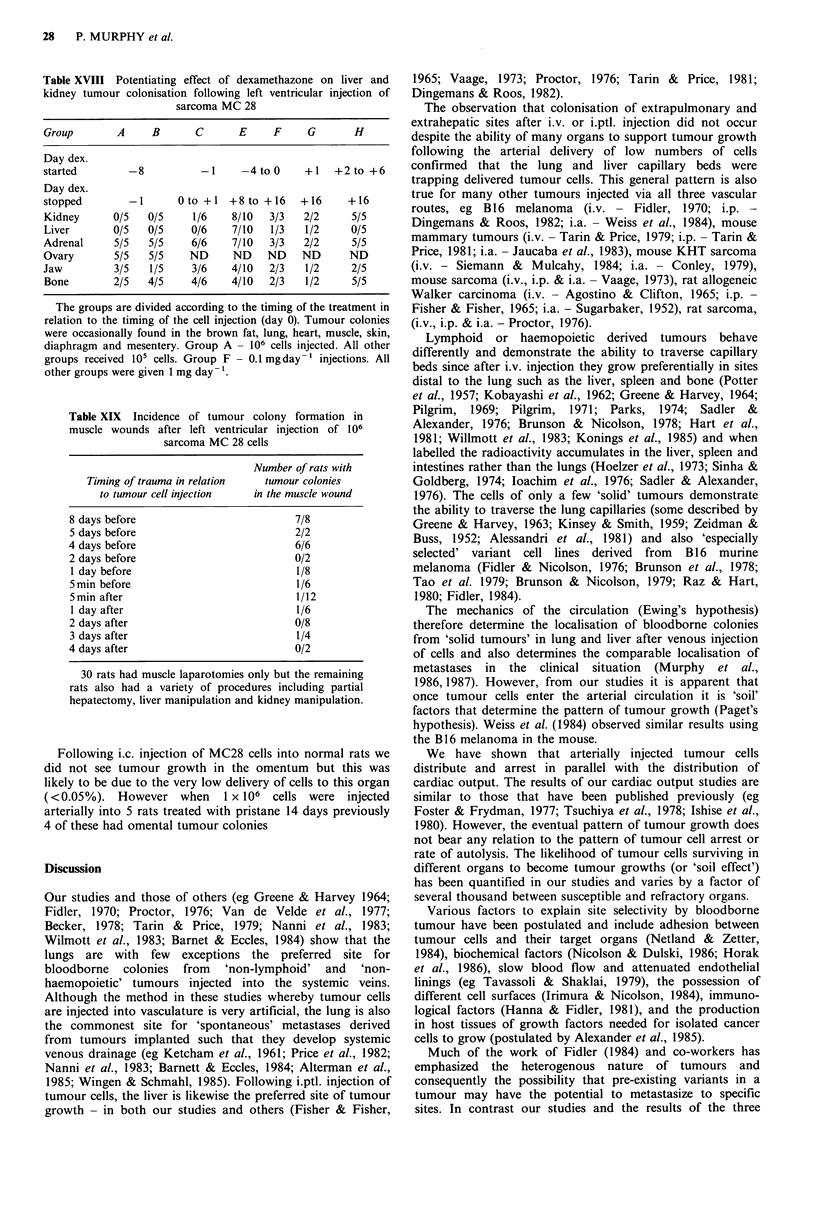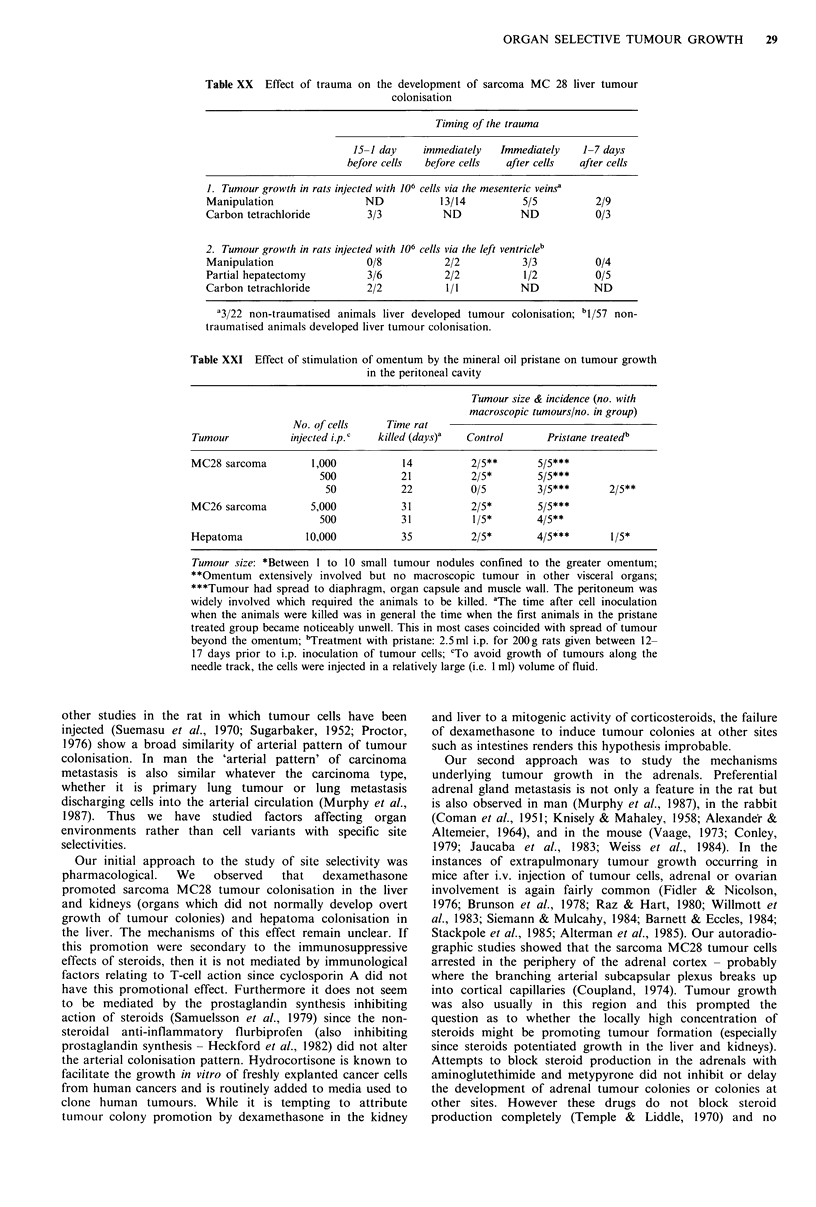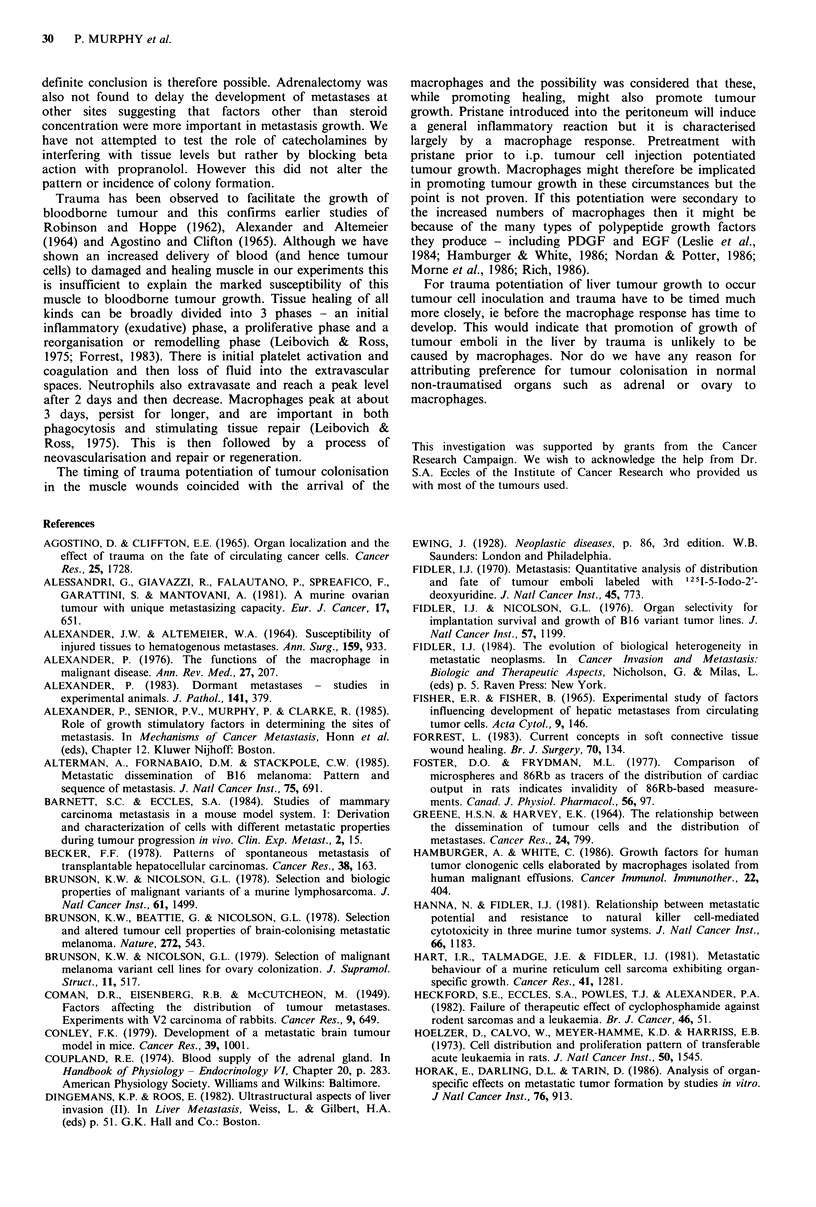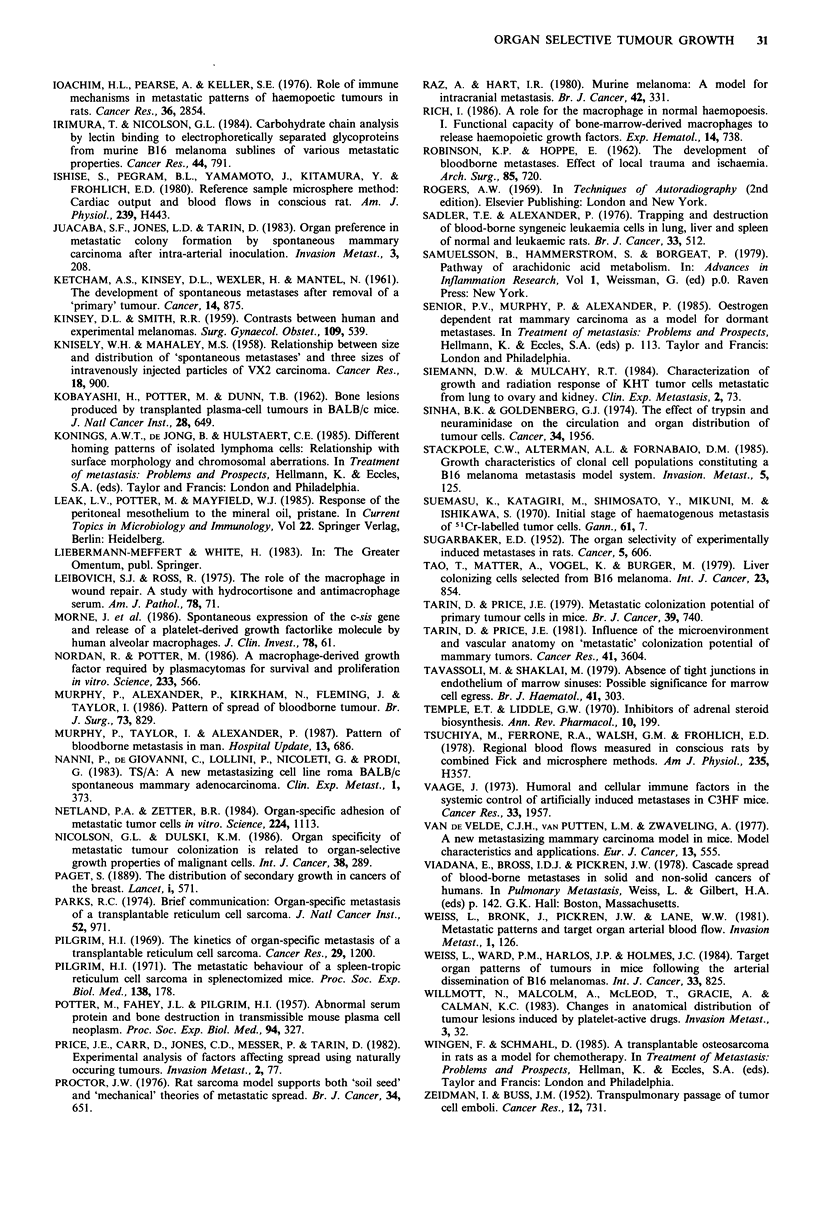# Mechanisms of organ selective tumour growth by bloodborne cancer cells.

**DOI:** 10.1038/bjc.1988.3

**Published:** 1988-01

**Authors:** P. Murphy, P. Alexander, P. V. Senior, J. Fleming, N. Kirkham, I. Taylor

**Affiliations:** University Surgical Unit, Southampton General Hospital, UK.

## Abstract

The sites of tumour development for 6 rat tumours injected into syngeneic rats via different vascular routes was determined. Xenografts of human tumours were also injected intra-arterially (i.a.) into immunosuppressed rats. Following intravenous (i.v.) and intraportal (i.ptl.) injection of cells tumour colonies localized in lung and liver respectively due to tumour cell arrest. Arterially injected radiolabelled cells disseminated and arrested in a similar distribution to cardiac output and did not 'home' to any organs. Following arterial injection of unlabelled tumour cells colonies grew in many organs. While the pattern of growth for a particular tumour varied with the cell dose, the 'arterial patterns' for all of the tumours studied followed a similar pattern. Some organs (eg adrenals, ovaries and periodontal ligament) were consistently preferred, others (eg skin and skeletal muscle) only supported tumour growth following the delivery of large numbers of cells, while in some tissues (eg spleen and intestines) tumour never grew. Viable tumour cells could be demonstrated by bioassay in many organs for up to 24h after i.a. injection. However tumour growth only occurred in certain organs and the pattern of this growth was not related to the number of tumour cells arrested or their rate of autolysis. This site preference could be expressed quantitatively as the probability of an arrested cell developing into a tumour and was considered a 'soil effect'. Site preference was not directly related to organ vascularity. Organ colonisation was promoted by steroid treatment but the mechanism was unclear and was not secondary to T-cell immunosuppression or prostaglandin synthesis suppression. The adrenal glands were preferred sites of tumour growth but pharmacological manipulation of adrenal function did not alter tumour growth to this organ. Sites of injury and healing were preferred sites of tumour colonisation and this could not be accounted for by increased delivery of tumour cells to these regions. The possibility that the macrophage component of the inflammatory response promoted tumour growth was suggested from studies in which the interval between trauma and inoculation of tumour cells was varied as well as by promotion of intraperitoneal (i.p.) tumour growth by a macrophage infiltrate.


Br. J. Cancer (1988), 57, 19–31 © The Macmillan Press Ltd., 1988

### Mechanisms of organ selective tumour growth by bloodborne cancer cells

P. Murphy[1], P. Alexander[2], P.V. Senior[2], J. Fleming[3], N. Kirkham[4]* & I. Taylor[1]

[1]*University Surgical Unit;* [2]*CRC Medical Oncology Unit;* [3]*Department of Nuclear Medicine; and* [4]*Department of Histopathology, Southampton General Hospital, Tremona Road, Southampton SO9 4XY, UK.*

**Summary** The sites of tumour development for 6 rat tumours injected into syngeneic rats via different vascular routes was determined. Xenografts of human tumours were also injected intra-arterially (i.a.) into immunosuppressed rats.

Following intravenous (i.v.) and intraportal (i.ptl.) injection of cells tumour colonies localised in lung and liver respectively due to tumour cell arrest. Arterially injected radiolabelled cells disseminated and arrested in a similar distribution to cardiac output and did not 'home' to any organs. Following arterial injection of unlabelled tumour cells colonies grew in many organs. While the pattern of growth for a particular tumour varied with the cell dose, the 'arterial patterns' for all of the tumours studied followed a similar pattern. Some organs (eg adrenals, ovaries and periodontal ligament) were consistently preferred, others (eg skin and skeletal muscle) only supported tumour growth following the delivery of large numbers of cells, while in some tissues (eg spleen and intestines) tumour never grew.

Viable tumour cells could be demonstrated by bioassay in many organs for up to 24 h after i.a. injection. However tumour growth only occurred in certain organs and the pattern of this growth was not related to the number of tumour cells arrested or their rate of autolysis. This site preference could be expressed quantitatively as the probability of an arrested cell developing into a tumour and was considered a 'soil effect'. Site preference was not directly related to organ vascularity. Organ colonisation was promoted by steroid treatment but the mechanism was unclear and was not secondary to T-cell immunosuppression or prostaglandin synthesis suppression. The adrenal glands were preferred sites of tumour growth but pharmacological manipulation of adrenal function did not alter tumour growth to this organ. Sites of injury and healing were preferred sites of tumour colonisation and this could not be accounted for by increased delivery of tumour cells to these regions. The possibility that the macrophage component of the inflammatory response promoted tumour growth was suggested from studies in which the interval between trauma and inoculation of tumour cells was varied as well as by promotion of intraperitoneal (i.p.) tumour growth by a macrophage infiltrate.

In explaining the mechanism of site selectivity by bloodborne metastasis the haemodynamic theory of Ewing (1928) stressed the importance of the mechanics of the circulation whereas the Soil/Seed hypothesis of Paget (1889) emphasized the importance of the environment in which the trapped emboli found themselves. The eventual sites of metastasis are, however, determined by both haemodynamic and soil/seed factors. Haemodynamic factors are important following the venous discharge of tumour cells since the 'organs of first encounter' are the commonest site for bloodborne metastasis – the lung after systemic venous discharge of tumour cells and the liver after portal venous discharge (Viadana *et al.*, 1978). The present study emphasizes that the 'soil' becomes important when tumour cells have entered the systemic arterial circulation and are thereby delivered to all organs. Clinically this situation can occur when cells are released from primary or secondary lung tumours or possibly when cells released into the venous circulation manage to traverse the lung capillaries.

In this paper we extend earlier data (Murphy *et al.*, 1986) on the pattern of spread of rat syngeneic non-lymphoid and non-haemopoietic tumours following intravenous (i.v.), intraportal (i.ptl.) and intra-arterial (i.a.) injection. In our i.v. and i.ptl. studies as well as previously reported studies tumour growth is essentially limited to the 'organs of first encounter' and the study of mechanisms of site selectivity is therefore limited. In order to deliver tumour cells to all organs we have injected them intra-arterially via the intracardiac (i.c.) route. Labelled cell distribution after i.c. injection has been compared with the distribution of cardiac output (measured using the microsphere technique) in order to establish whether cells disseminate and arrest passively or whether they recirculate and localise in certain organs. The 'arterial pattern' of tumour growth after unlabelled cell injection has then been compared with the arterial distribution of labelled tumour cells to establish and quantitate the 'soil effect'.

Having established a 'soil effect' after arterial injection of tumour cells we have attempted to determine the mechanisms using several approaches. Firstly a correlation of site selectivity with organ vascularity (using data obtained from the microsphere studies) was sought since there have been reports that more vascular organs might be more susceptible to metastasis (Weiss *et al.*, 1981). Secondly the rate of autolysis of trapped labelled cells in different organs has been compared with the organs susceptibility to tumour growth. Thirdly we have intervened pharmacologically to see if site preference can be altered and finally we have rendered refractory organs susceptible to tumour growth by trauma.

The pharmacological studies took two directions. Because steroids promoted sarcoma and hepatoma tumour growth in liver and kidney possible steroid dependence of the sarcoma was looked for by testing if adrenalectomy inhibited tumour growth. To establish if the steroid potentiating effect was related to T-cell immunosuppression or inhibition of prostaglandin synthesis the animals were treated with cyclosporin A or a non-steroidal anti-inflammatory agent flurbiprofen.

As the adrenal gland in our studies was found to be a universally preferred site of tumour colonisation the possibility that locally high steroid concentrations in the adrenal provided an environment conducive to tumour growth as examined by testing if the steroid inhibitors metyrapone and aminoglutethimide inhibited tumour growth in the adrenal. We did not test the role of catecholamines in adrenal preference by interfering with it's tissue levels but instead studied the effect of blocking the beta action of catecholamines with propranolol.

We found that sites of trauma or healing were especially susceptible to tumour growth. The possibility that this was related to increased bloodflow and hence increased delivery

*Present address: Department of Histopathology, Royal Sussex Hospital, Brighton, UK.
Correspondence: P. Alexander.
Received 9 June 1987; and in revised form, 2 October 1987.

of tumour cells was eliminated using the microsphere method. The possibility that it was related to the macrophage response of the inflammatory process was then studied in an intraperitoneal (i.p.) system. The omentum was found to be very susceptible to tumour growth after i.p. injection of tumour cells. This organ is very rich in monocytes and macrophages (Liebermann-Meffert & White, 1983) and following local administration of the branched chain hydrocarbon pristane there is a dramatic enlargement of the omentum (Leak *et al.*, 1985) and production of a exudate rich in macrophages which, however, are not cytocidal (Alexander, 1976). If macrophages were responsible for promoting the growth of cancer cells at sites of inflammation or injury then pretreatment with pristane should facilitate the growth of i.p. tumour cells.

### Materials and methods

### Animals

The studies employed syngeneic hooded Lister rats which were initially obtained from the Institute of Cancer Research at Sutton. Males weighed 200–330 g and females 150–250 g.

### Anaesthesia

The animals were anaesthetised using open ether.

### Injections

I.v. injections were made through cannulae placed in the right jugular vein. I.ptl. vein injections were made directly into the portal vein or alternatively into one of the mesenteric ileal tributaries of the portal vein.

I.a. injections were made through cannulae (Portex 800/100/100) inserted retrogradely via the right carotid artery into the left ventricle of the ether anaesthetized rat. In early studies the animals were allowed to wake and were then injected. In later studies, when cannulation was performed more rapidly, tumour cells were injected into anaesthetised rats at the time of cannulation and this became the preferred method of injection.

### Tumours

Six chemically induced syngeneic to the hooded Lister rat were used in the intravascular experiments (Table I). A seventh tumour, the methylcholanthrene induced sarcoma MC26, was only used in the intraperitoneal experiments. Six tumours had been induced at the Chester Beatty Institute of Cancer Research and one in Southampton (the breast carcinoma – Senior *et al.*, 1985). They were passaged s.c. and the passage number was not observed to affect the growth pattern of the tumours after i.v. injection.

Human tumours were also injected i.a. into immuno-suppressed rats. These tumours had all previously been passaged in athymic nude mice. Melanoma HX34 and carcinoma HX70 was passaged in cyclosporin A treated rats (given s.c. at a dose of 25 mg kg$^{-1}$ four times per week) prior to i.c. injection into further cyclosporin A treated rats. Melanoma HX46 was removed from the nude mouse, disaggregated and injected straight into nude hooded Lister rats.

### Tumour cell suspensions

These were prepared by enzymatic disaggregation of the tumour fragments augmented with a magnetic stirrer (Hank's basic salt solution containing 100 $\mu$g ml$^{-1}$ of bacterial neutral protease and 1 $\mu$g ml$^{-1}$ of DNAase was used). Following washing three times in Hank's (centrifuged at 900 rpm), the cells were either immediately injected or alternatively they were cultured for a total of 36–48 h before injection – cultured in medium made up from 89% MEM Eagles medium (containing 5% sodium bicarbonate 7.5%, 1% MEM sodium pyruvate, 1% non-essential amino acids, 1% penicillin/streptomycin solution), 10% foetal calf solution and 1% glutamine. The medium was changed 16–24 h after the cells had first been put in the flasks. Cells were then harvested from the flasks 16–24 h later using the same enzymatic preparation and washing methods as above. The preparations from cultured cells gave suspensions which were almost entirely made up of single cells whereas preparations made directly from the tumour frequently contained clumps of cells. Cell counting was done in an improved Neubauer counting chamber. The hepatoma and sarcomas grew *in vitro* and could be radiolabelled but this did not apply to the breast carcinoma. Cell viability for the sarcomas, assessed using trypan blue exclusion, was between 80% and 97% (though in some instances deliberately poorer cell preparations were used – see below), and for the hepatoma was 70%–80%.

### Radiolabelling of the tumour cells

The cells were cultured as above, but at the time the media were changed, 5-[$^{125}$I]Iodo-2′-deoxyuridine (IUdr) was added to make a concentration of ~0.2 mCi ml$^{-1}$. Cytospin preparations of the labelled sarcoma cells were autoradiographed and this confirmed that over 90% of the tumour cells had taken up the IUdr.

### Organ counts

Alcohol extraction of free iodide was performed by the addition of 70% alcohol to the minced organs and was replaced every day for 3 days. The organs were counted in a Wallac Decem series automatic well gamma counter. The carcass (ie that which remained after removal of the organs) and skin were counted by placing them 18 cm under a lead

**Table I** Syngeneic rat tumours used

| *Tumour* | *Passages used* | *Induced by* | *Comments* |
|---|---|---|---|
| Sarcoma MC28 | 27–32 | Methylcholanthrene | Low immunogenicity. Spontaneous metastases |
| Sarcoma MC24 | 10–13 | Methylcholanthrene | High immunogenicity. |
| Sarcoma HRGCll | 4–5 | Dimethylhydrazine | Both were induced in attempts to produce |
| Sarcoma HRGC9 | 2–4 | Dimethylhydrazine | colonic tumours. |
| Hepatoma | 130–142 | Dimethylaminoazobenzene | Anaplastic |
| Breast carcinoma OES5 | 5–18 | Oestrogen | Oestrogen dependent. Grew very slowly in absence of exogenous s.c. oestrogen pellet. |

shielded gamma camera with a 33 cm diam. crystal. Radioactivity counts from iodine-125 were partially absorbed by the carcass of the rat. For the average rat carcass of ~130 g, absorption was ~13% and all carcass counts were corrected to allow for this. Using a standard, the carcass and skin values were then converted to the same scale as the organ values.

To determine 'arterial' distribution of labelled tumour cells ~$1 \times 10^6$ labelled sarcoma MC28 or hepatoma cells were injected in each rat. The rats were killed with pentobarbitone forte given i.v. Since the number of cells injected varied by up to 20% between animals, the results for the immediate and 5 min distributions were expressed by summing the total radioactivity counts to 100% and then the individual organ values were expressed as fractions of this value. For the later time points the organs were 'alcohol extracted'. The counts of the organs that could not be alcohol extracted (ie carcass, testis and pancreas for technical reasons and bladder and skin because of contamination with iodine) were excluded. The remaining organ counts were totalled to 100% and a 'relative cell distribution' was thereby obtained.

*Autoradiography*

Autoradiography (as described by Rogers, 1969) was also used to study cytospin labelled tumour cell preparations and organ sections following vascular injection of labelled cells.

*Identification of tumour colonies*

The rats were usually killed when the first member of a group became unwell. The rats were skinned and all sites except the brain were examined for overt metastases. Frequent histological examination was made to confirm the absence or presence of growth in all organs. Bony growths were most often seen in the ribs. Vertebral growths caused the rats to develop early paraplegia and their presence was confirmed by X-radiography.

*Bioassay of organs for the presence of viable tumour cells or micrometastases*

Organs without overt metastasis were bioassayed for the presence of microscopic colonies or trapped viable cells (cf. Alexander, 1983) by i.p. transplantation of the 'minced' organ to recipient rats. If these contained viable cells they then grew out in the peritoneum of the recipient.

*Distribution of cardiac output*

This was determined by the left ventricular injection of ~60,000–100,000 Co-57-labelled styrene divinyl benzene copolymer microspheres into each rat ($16.4 \pm 0.5\,\mu m$ – supplied by New England Nuclear). These distribute and arrest in the organs in the same proportions as the percentage distribution of cardiac output (Ishise *et al.*, 1980). The percentage radioactivity in each removed organ following injection represents a direct measure of the distribution of cardiac output. Radioactivity counts in the organs (and the converted carcass and skin counts) were totalled and taken as the '100% value'. Radioactivity from Co-57 (unlike that from I-125) was not significantly attenuated by the carcass and therefore no correction was needed.

*Calculation of the 'relative vascularity' of the rat's organs*

The vascularity of an organ is usually expressed as ml blood $min^{-1} g^{-1}$ of organ and can be determined from the cardiac output per minute, the percentage distribution of cardiac output to the organ in question, and the weight of the organ. Since in our studies only the fractional distribution of cardiac output and organ weight were measured and not the cardiac output per minute, a 'relative vascularity' has been determined by dividing percentage of cardiac output by the percentage weight of the organ in each individual rat.

*Drugs used in the rats and other procedures undertaken*

In order to study some the factors that might determine the site of growth of metastases, dexamethasone, aminoglutethimide, metyrapone, flurbiprofen and propranolol were used at dosages stated in the text. Additionally in these studies a number of rats underwent bilateral adrenalectomies and were maintained by the addition of normal saline to their drinking water.

*Induction of trauma*

Mechanical liver trauma was performed by squeezing the liver between two fingers across it's width while the rats were anaesthetized under ether. Partial 2/3 hepatectomies were performed by ligating the anterior lobes of the liver at their pedicles and excising them. Chemical liver trauma sufficient to cause later development of cirrhosis was induced by giving $0.5\,ml\,kg^{-1}$ of carbon tetrachloride once via a nasogastric tube. Muscle trauma was standardised by performing a routine midline longitudinal laparotomy scar and then suturing the abdominal musculature in one layer with linen. No wound infections were observed.

### Results

*Inability to observe recirculation of tumour cells*

Study of organ site selectivity by bloodborne tumour cells must initially establish how the tumour cells distribute and localise after entry into the vasculature. In our particular 'model' of vascular spread evidence from several sources shows that tumour cells mainly arrest on initial delivery to an organ and do not recirculate after injection via the different vascular routes used. After i.v. and i.ptl. injection tumour growth is almost completely localised to lung and liver respectively (Tables II and III). However, after i.a. injection tumour colonies form in many organs (Tables IV–VII). This demonstrates that the failure to cause systemic tumour growth following i.v. and i.ptl. inoculation is not because the cells cannot grow in distant organs but is due to the inability of significant numbers of cells to pass in a viable state through the capillaries of the lung and liver.

Following the i.v. and i.ptl. injection of radiolabelled tumour cells, radioactivity was essentially confined to the lung and liver respectively (Tables VIII–X). The small amount of radioactivity that was in other organs shortly after i.v. injection comes from labelled debris and dying cells contained in the inoculate. This was demonstrated by the i.v.

**Table II** Pattern of tumour colonisation following the intravenous injection of unlabelled tumour cells

| *Tumour type and (No. of cells)* | *No. of rats with lung tumours* | *No. of rats developing tumours at distant sites*[a] |
|---|---|---|
| MC28 sarcoma ($1 \times 10^3$–$2 \times 10^6$) | 37/51 | 0/51 |
| MC24 sarcoma (1 to $5 \times 10^6$) | 3/3 | 0/3 |
| Breast carcinoma ($1 \times 10^3$–$1 \times 10^6$) | 16/19 | 0/19 |
| Hepatoma ($1 \times 10^6$) | 4/4 | 0/4 |
| HRGCll sarcoma ($1 \times 10^6$) | 6/6 | 0/6 |

[a]At times when animals became unwell from lung lesions (see **Materials and methods**).

**Table III** Distribution of tumour colonies in rats after intraportal vein injection of tumour cells

| *Tumour type (No. of cells)* | *No. of rats with liver tumour colonies* | *No. of rats with lung tumour colonies* | *No. of rats with distant tumour colonies* |
|---|---|---|---|
| Sarcoma MC28 ($1 \times 10^3$–$1 \times 10^6$) | 45/88 | 1/88 | 0/88 |
| Hepatoma ($5 \times 10^5$–$1 \times 10^6$) | 11/13 | 2/13 | 0/13 |
| Breast carcinoma ($1 \times 10^6$) | 10/10 | 2/10 | 0/10 |

Cultured and directly disaggregated cell preparations included.

**Table IV** Sites of overt tumour colonisation in rat organs after left ventricular injection of sarcoma MC28 at different doses. All cells cultured for ~36–48 h

| *No. cells* | *1–4 × $10^6$* | *$10^5$* | *$10^4$–3 × $10^4$* | *$10^3$* | *$10^2$* |
|---|---|---|---|---|---|
| No. of rats with tumours | 57/57 | 29/30 | 12/13 | 10/15 | 0/5 |
| No. injected conscious. | 22/57 | 15/30 | 13/13 | 15/15 | 5/5 |
| Day killed[f] | | | | | |
| Median | 15 | 19 | 25 | 32 | – |
| Range | 11–21 | 15–23 | 22–32 | 26–39 | – |
| Adrenals | 57/57 (0/57) | 29/30 (1/29) | 9/13 (1/9) | 2/15 (2/2) | 0/5 |
| Ovaries | 13/14 (1/13) | 8/11 (5/8) | 8/9 (2/8) | 2/10 (2/2) | 0/5 |
| Brown fat[d] | 22/22 | 14/15 | 10/13 | 7/15 | 0/5 |
| Brown fat[e] | 19/35 | 2/15 | ND | ND | ND |
| Bone | 33/57 | 19/30 | 7/13 | 6/15 | 0/5 |
| Periodontal ligament | 32/40[c] | 12/23[c] | 1/13 | 1/15 | 0/5 |
| Lungs | 19/57 | 3/30 | 0/13 | 2/15[a] | 0/5 |
| Diaphragm | 11/57 | 0/30 | 0/13 | 0/15 | 0/5 |
| Muscle | 9/38[c] | 1/17[c] | 1/13[a] | 1/15[a] | 0/5 |
| Skin | 10/38[c] | 5/17[c] | 0/13 | 0/15 | 0/5 |
| Heart | 2/57 | 2/30 | 0/13 | 0/15 | 0/5 |
| Mesentery | 5/57 | 6/30 | 0/13 | 0/15 | 0/5 |
| Yellow fat | 8/57 | 9/30 | 0/13 | 1/15[a] | 0/5 |
| Pancreas | 2/57 | 0/30 | 0/13 | 0/15 | 0/5 |
| Liver | 1/57 | 0/30 | 0/13 | 0/15 | 0/5 |
| Kidneys[b] | 0/57 | 0/30 | 0/13 | 0/15 | 0/5 |
| Intest. | 0/57 | 0/30 | 0/13 | 0/15 | 0/5 |
| Spleen | 0/57 | 0/30 | 0/1 | 0/15 | 0/5 |
| Testis | 0/43 | 0/19 | 0/4 | 0/5 | ND |
| Thymus | 0/57 | 0/30 | 0/13 | 0/15 | 0/5 |

( ) Indicates the frequency with which the adrenals and ovaries developed unilateral metastasis as opposed to both organs being involved; [a]Single metastases in each core; [b]These develop microscopic metastases; [c]Not all animals were examined for metastases at these sites; [d]Conscious rats; [e]Anaesthetized rats; [f]Only rats developing metastases included.

injections of deliberately damaged cell preparations which led to high fractions of radioactivity localising to the liver while after the injection of a cell preparation cleared of debris the radioactivity in the liver was low (Table XI). The debris is presumably cleared by the reticuloendothelial system. Hepatoma cell preparations contained a greater proportion of non-viable cells which probably explains the higher fraction in the liver after i.v. injection (Table IX). With time radioactivity accumulates in other organs (Table VIII) but this cannot be due to the release of intact cells from the lungs into the arterial circulation since the pattern of radioactivity seen was not the same as that following injection of labelled cells into the left ventricle.

There were three lines of evidence to suggest that tumour cells do not recirculate significantly after arterial injection. Firstly, the labelled tumour cell distribution (values shown for 5 min after i.a. injection) paralleled the distribution of cardiac output in conscious and anaesthetised rats as shown in Tables XII and XIII. The cells must have been carried passively in the blood stream and then arrested on 'first pass'. The only exceptions are that the cell associated radioactivity in the liver and lung was much higher than the proportion of cardiac output to lung and liver. The raised liver values was in part secondary to clearance of labelled debris (as already seen after i.v. injection) since injection of low viability cell preparations resulted in high clearances by the liver (Table XI). There is probably true transcapillary passage of some 15% of the cells across the gastro-intestinal capillary beds to the liver and across the systemic capillary beds (mainly skeletal muscle) to the lungs. This occurs within the first 5 min after injection and is not significant in terms of tumour cell recirculation since these cells then arrest in liver and lung.

Secondly, the consistently higher value for cardiac output distribution to brown fat seen in conscious rather than anaethetised rats was reflected both in the increased distribution of labelled tumour cells to brown fat in conscious rats (Tables XII and XIII) and the higher frequency of development of tumour colonies in this organ in conscious rats (eg as in Table IV) ie further confirming the passive nature of tumour cell distribution and arrest on 'first pass'. Thirdly the experiments in Table XIV show that

**Table V** Frequency of overt colonisation in female rat organs after left ventricular injection of breast carcinoma at different doses

| | *Number of cells injected* | | | |
|---|---|---|---|---|
| | $3\times10^5$–$10^6$ | $10^5$ | $10^4$ | $10^3$ |
| Number of rats developing metastases | 47/48[a] | 3/3 | 4/4 | 0/3 |
| No. injected conscious | 4/48 | 0/3 | 0/4 | 0/3 |
| Adrenals | 46/48 (0/46) | 3/3 (0/3) | 3/4 (3/3) | 0/3 |
| Ovaries | 44/48 (2/46) | 3/3 (1/3) | 4/4 (1/4) | 0/3 |
| Bone | 10/48 | 1/3 | 2/4 | 0/3 |
| Periodontal ligament | 14/48 | 2/3 | 1/4 | 0/3 |
| Lungs | 29/48 | 3/3 | 4/4 | 0/3 |
| Brown fat conscious | 1/4 | ND | ND | ND |
| Brown fat anaesthetised | 3/44 | 0/3 | 0/4 | 0/3 |
| Diaphragm | 1/48 | 0/3 | 0/4 | 0/3 |
| Skin | 3/18 | 0/3 | 0/4 | 0/3 |
| Heart | 2/48 | 0/3 | 0/4 | 0/3 |
| Kidneys | 2/48 | 1/3 | 0/4 | 0/3 |
| Liver | 2/48 | 0/3 | 0/4 | 0/3 |
| Uterus | 2/48 | 0/3 | 0/4 | 0/3 |
| Peritoneum | 3/40 | 0/3 | 0/4 | 0/3 |
| Muscle | 0/18 | 0/3 | 0/4 | 0/3 |

No tumour developed in the intestines, spleen or thymus. ( ) indicates the number of times the adrenals and ovaries developed unilateral tumour growths as opposed to both organs being involved; [a]Preoestrogenised, late oestrogenised and non-oestrogenised rats were used. Time of oestrogenisation did not alter the pattern colonisation but affected the rate of appearance of colonies.

Distribution of tumour colonisation in male rats after left ventricular injection of tumour cells

Oestrogenised and non-oestrogenised animals all included. $10^6$ cells injected into 13 male rats. All rats developed metastasis

| | |
|---|---|
| Adrenal | 11/13 (unilateral in 1) |
| Lung | 8/13 |
| Muscle, diaphragm, jaw | 3/13 |
| Bone, skin, heart, kidney | 1/13 |

**Table VI** Frequency of tumour colonisation in rat organs after left ventricular injection of hepatoma cells at different doses

| | *Number of cells injected* | |
|---|---|---|
| | $3\times10^5$–$10^6$ | $10^5$ |
| No. of rats developing metastases | 13/13 | 3/3 |
| Adrenals | 13/13 (3/13) | 2/3 (1/2) |
| Ovaries | 4/4 (1/4) | ND |
| Bone | 4/13 | 3/3 |
| Periodontal ligament | 2/6 | 0/3 |
| Eyes | 2/13 | 0/3 |
| Kidneys | 1/13 | 0/3 |

Cultured cells in 7 animals. Directly disaggregated in the remainder. Tumour colonies were not seen in the lungs, liver, intestines, spleen, pancreas, mesentery, diaphragm, testes and thymus. Only 3 of the high dose group of animals were examined for muscle and skin metastases – none were seen. () Incidence of tumour growth occurring unilaterally.

the rat's blood was 'bioassay negative' for tumour cells 5 min after i.a. injection. This again demonstrates the rapid clearance of tumour cells from the blood.

*Tissue preference following arterial injection ('Soil effect'): General pattern and quantitation*

Comparison of arterial tumour cell distribution (eg for sarcoma and hepatoma – see Tables XII and XIII) with the eventual pattern of tumour growth (see Tables IV and VI) shows no correlation ie tumour colonisation is site selective. The overall pattern of tumour growth for all the tumours studied (Tables IV–VII) show that they have similar site preferences. Organs such as the adrenals, ovaries, periodontal ligament and bone were consistently preferred sites of colonisation, others such as the pancreas, diaphragm, skin and skeletal muscle only developed tumour colonies (rarely more than 10 in skin or muscle) following the delivery of large numbers ($10^6$) of tumour cells, while others such as the intestines, spleen and testes never developed colonies even after the injection of $>10^6$ cells. Only tumour growth in the kidneys, brown fat and to a lesser degree the lungs varied depending on the tumour type injected. Human tumour cells injected into immunosuppressed rats (Table XV) tended to grow mainly in the susceptible organs (ie adrenals, bone and periodontal ligament).

Sarcoma MC28 tumour cells arrested mainly singly and widely separated from each other – of 100 radiolabelled cells examined in autoradiographic sections of the adrenal (the animals were killed 5 min after i.c. injection of $1\times10^6$ labelled cells), all were found in the outer cortex and 86% were single and 14% were found as two cells together. Of 100 cells examined in the kidneys, 94% were in the glomeruli and of these 84% were single. Since most cells trapped singly, and since the approximate percentage of cells delivered to each organ was known and the frequency of tumour colonisation for any particular cell dose was also known, the likelihood of a trapped tumour cell developing into a tumour colony could be calculated for individual organs. For example, 3 out of 4 rats developed adrenal tumour growths (though the number of individual growths could not be counted since they coalesced) following the

**Table VII** Frequency of tumour colonisation in rat organs after left ventricular injection of sarcomas MC24, HRGCll and HRGC9

| *Tumour* | *Sarcoma MC24* | *Sarcoma HRGCll* | *Sarcoma HRGC9* |
|---|---|---|---|
| Number of animals developing metastases | 10/10 | 9/9 | 10/14 |
| Sex | m | f | 10f, 4m |
| Anaesthetized | No | Yes | 9/14 |
| Number of cells | $10^6$–$2\times10^6$ | $10^6$ | $5\times10^5$–$10^6$ |
| Adrenals | 6/10 | 6/9 (3/6) | 9/14 (1/9) |
| Ovaries | – | 7/9 (3/7) | 5/14 (1/5) |
| Bone | 1/10 | 4/9 | 2/14 |
| Periodontal ligament | NE | 2/9 | 0/14 |
| Lung | 4/10 | 5/9 | 4/14 |
| Heart | 1/10 | 0/9 | 0/14 |
| Pancreas | 1/10 | 0/9 | 0/14 |
| Mesentery | 1/10 | 0/9 | 0/14 |
| Yellow fat | 1/10 | 1/9 | 1/14 |
| Skin | 1/7[a] | 3/9 | 3/14 |
| Muscle | 1/7[a] | 2/9 | 2/14 |
| Diaphragm | 0/10 | 0/9 | 0/14 |
| Intestines | 0/10 | 0/9 | 0/14 |
| Liver | 0/10 | 0/9 | 0/14 |
| Spleen | 0/10 | 0/9 | 0/14 |
| Thymus | 0/10 | 0/9 | 0/14 |
| Testes | 0/10 | – | 0/4 |
| Kidneys | 0/10 | 7/9[a] | 9/14 |
| Brown fat | 10/10 | 3/9[b] | 2/14 |

() Incidence of tumour colonisation occurring unilaterally; [a]multiple tumour colonies; [b]single tumour colonies. NE = not examined.

**Table VIII** Percentage distribution of labelled MC28 sarcoma cells after intravenous injection

| *Time after injection* | *Lungs* | *Liver* | *Kidneys* | *Carcass*[a] | *Total* |
|---|---|---|---|---|---|
| Immed. ($n=3$) | 93.08±2.80 | 1.51±0.32 | 0.14±0.03 | 5.27± 2.60 | 100% |
| 5 min ($n=12$) | 93.94±2.09 | 2.50±0.55 | 0.07±0.05 | 3.49± 1.54 | 100% |
| 4 h ($n=5$) | 40.57±8.08 | 4.75±0.57 | 0.61±0.21 | 27.89± 8.48 | 73.8% |
| 8 h ($n=4$) | 29.91±2.61 | 3.19±0.69 | 0.62±0.24 | 35.32±10.54 | 69.0% |
| 16–24 h ($n=4$) | 1.03±0.24 | 0.47±0.20 | 0.07±0.03 | 7.07± 1.75 | 8.6% |
| 48 h ($n=6$) | 0.13±0.12 | 0.42±0.08 | 0.04±0.01 | 5.45± 4.22 | 6.0% |

[a]Skin, bladder and thyroid were removed in the rats killed at the later time points because of iodine contamination. The immediate and 5 min results are calculated by the addition of all the organ counts and calling the total 100%. This assumes none of the injected radioactivity has been excreted. The values at the remaining time points have been calculated from an estimate of the amount of radioactivity injected.

**Table IX** Early percentage distribution of labelled hepatoma cells after intravenous injection

| | *% of tumour cells trapping immediately after i.v. injection (n=4) %±s.d.* | *% of tumour cells trapping 5 min after i.v. injection (n=3) %±s.d.* |
|---|---|---|
| Lungs | 87.42±4.71 | 82.57±2.20 |
| Liver | 6.90±3.99 | 12.56±1.07 |
| Kidneys | 0.18±0.12 | 0.22±0.11 |
| Adrenals | 0 | 0 |
| Carcass and remaining organs | 5.5±0.86 | 4.65±1.36 |

injection of $10^4$ sarcoma MC28 cells ie following the approximate delivery of 30 tumour cells to both adrenals. Therefore ~1 in 30 cells arresting in the adrenals gave rise to a tumour colony. The figures are similar for the periodontal ligament and the ovaries. In contrast, over a thousand times that number of cells arresting in the intestines or over a hundred times that number arresting in the spleen (and still 'bioassay positive' 24 h later – see Table XIV) failed to develop into tumour colonies.

*Mechanisms for site selectivity*

*Rate of autolysis of cancer cells trapped in the capillaries beds of different organs* Autolysis of labelled tumour cells following i.v. injection (Table VIII) revealed the disappearance of most of the label from the lung and release into tissue fluids over a few hours. By 8 h 70% of the radioactivity had disappeared and by 24 h 99% had disappeared. The rate of autolysis over 16 h following i.a. injection of sarcoma MC28 was also studied and was not greater in those organs refractory to the growth of tumour emboli than those which are susceptible (Table XVI). The 'soil effect' cannot therefore be simply explained in terms of the rate at which the trapped cells die within the capillaries.

*Relationship to organ vascularity* The possibility that site selection might be directly related to organ vascularity was

**Table X** Percentage distribution of radioactivity following intraportal injection of labelled sarcoma MC28 cells and hepatoma cells

| | *Sarcoma MC28* | | *Hepatoma* | |
|---|---|---|---|---|
| | *Immediately after inj. (n=3) %±s.d.* | *5 min after inj. (n=3) %±s.d.* | *Immediately after inj. (n=3) %±s.d.* | *5 min after inj. (n=3) %±s.d.* |
| Liver | 95.35±1.34 | 95.86±0.97 | 95.10±2.15 | 97.32±2.13 |
| Lungs | 0.30±0.04 | 0.27±0.12 | 0.79±0.84 | 0.14±0.06 |
| Kidneys | 0.10±0.06[a] | 0.08±0.03[a] | 0.09±0.04 | 0.06±0.05 |
| Carcass | 4.25±1.31 | 3.79±1.10 | 4.02±1.38 | 2.48±2.07 |

[a]Only the right kidney counted in these cases. The organ counts were totalled to make 100% and the percentages were then calculated.

**Table XI** The effect of cell viability on clearance of radioactivity by the liver after intravenous injection of sarcoma MC28 cells

| | *% ±s.d. of radioactivity in the liver 5 min after injection* | |
|---|---|---|
| | *After i.v. injection (n=3)* | *After i.c. injection (n=3)* |
| 97% viable[a] | 0.63±0.07[b] | ND |
| Over 80% viable | 7.59±0.69 | 13.47±1.50 |
| Less than 80% viable[b] | 12.49±1.02 | 20.42±0.33 |

[a]Cells separated from debris on a Ficoll gradient and then washed twice in Hanks before injection; [b]Cells allowed to stand for 4 h at high density during which time the viability decreased.

studied. Although there was a degree of correlation between susceptibility to tumour colonisation and high organ vascularity (Table XVII) there were exceptions such as kidney and heart which were very vascular but not nearly as susceptible to tumour colonisation as adrenals and ovary. Measurements of vascularity are however difficult to interpret since they are likely to be variable and also many organs are not uniform vascular structures (eg the adrenal or kidney – each with a cortex and medulla).

*Effect of steroids, cyclosporin A and flurbiprofen* The liver did not normally support tumour growth after i.a. injection of sarcoma MC28 or hepatoma cells and no microscopic tumour growths could be demonstrated histologically. However the steroid dexamethasone given s.c. was found to promote the growth of sarcoma MC28 liver tumour colonies if given within 24 h of tumour cell injection (Table XVIII) and also in 5 out of 7 rats given $10^6$ hepatoma cells i.a. In the kidneys microscopic glomerular tumour deposits were routinely observed after i.a. injection of $10^6$ sarcoma MC28 cells. Although viable cells could be demonstrated on bioassay these growths did not develop into macroscopic lesions. However if dexamethasone was given at the time of tumour injection or as long as two days afterwards large overt tumour growths developed (Table XVIII).

Attempts were made to ascertain why steroid treatment potentiated the growth of tumour colonies. Sarcoma MC28 tumour cells injected into animals bilaterally adrenalectomised on day −8, or day +7, or at the same time as the cancer cells were injected to see if the tumour was steroid dependent but there was no inhibition of the growth of tumour in any of the susceptible organs of these rats (data not shown). To see if tumour potentiation by the steroids was mediated by T-cell immunosuppression or by the inhibition of prostaglandin synthesis the sarcoma cells were injected into six rats treated with cyclosporin A given s.c. at 25 $mg\,kg^{-1}$ four times weekly commencing one day before tumour inoculation or flurbiprofen (given 7 $mg\,kg^{-1}$ twice daily also commencing on day −1 – see Heckford *et al.*, 1982) respectively. Neither of these treatments caused the development of tumour colonies at lung or liver or altered the pattern of growth in other organs (data not shown).

**Table XII** Comparison of the % distribution of cardiac output with the % distribution of tumour cells 5 min after injection into conscious rats

| | | *% Distribution cardiac output* | *% Distribution sarcoma cells* | *% Distribution hepatoma cells* |
|---|---|---|---|---|
| Number of rats | m. | 7 | 11 | 4 |
| | f. | 9 | ND | ND |
| Carcass[a] | m. | 40.92±7.23 | 30.28±6.89 | 21.19±3.88 |
| | f. | 44.50±7.78 | ND | ND |
| Kidneys | m. | 15.12±4.31 | 8.95±2.83 | 8.29±3.66 |
| | f. | 11.53±3.76 | ND | ND |
| Heart | m. | 7.93±3.30 | 5.66±1.90 | 2.69±1.07 |
| | f. | 5.87±2.27 | ND | ND |
| Skin | m. | 9.33±2.51 | 6.72±3.38 | 4.67±1.46 |
| | f. | 5.05±4.69 | ND | ND |
| Brown fat | m. | 4.62±2.45 | 5.98±2.53 | 3.99±0.97 |
| | f. | 10.12±4.36 | ND | ND |
| Brain | m. | 3.20±0.75 | 1.57±0.79 | 0.99±0.52 |
| | f. | 2.39±1.07 | ND | ND |
| Adrenals | m. | 0.39±0.20 | 0.19±0.09 | 0.18±0.10 |
| | f. | 0.34±0.12 | ND | ND |
| Testes | m. | 1.19±0.63 | 0.83±0.45 | 0.89±0.39 |
| Ovaries | f. | 0.17±0.15 | ND | ND |
| Lungs | m. | 1.05±0.40 | 17.33±5.99 | 24.45±6.12 |
| | f. | 0.75±0.51 | ND | ND |
| Pancreas | m. | 2.14±1.18 | 1.06±0.44 | 1.26±0.53 |
| | f. | 2.45±0.94 | ND | ND |
| Spleen | m. | 0.78±0.38 | 0.61±0.54 | 0.92±0.64 |
| | f. | 0.62±0.27 | ND | ND |
| Small bowel | m. | 6.09±1.56 | 4.29±1.03 | 3.25±1.47 |
| | f. | 8.85±2.67 | ND | ND |
| Large bowel | m. | 3.02±0.96 | 1.68±0.38 | 1.26±0.30 |
| | f. | 3.35±1.30 | ND | ND |
| Stomach | m. | 2.05±1.10 | 0.95±0.25 | 1.09±0.27 |
| | f. | 1.98±1.02 | ND | ND |
| Mesentery | m. | 1.00±0.35 | 0.70±0.25 | 0.76±0.52 |
| | f. | 0.87±0.37 | ND | ND |
| Liver | m. | 0.36±0.32 | 13.59±2.85 | 23.78±6.39 |
| | f. | 0.59±0.48 | ND | ND |
| *Organs not measured in all animals. () No. of rats* | | | | |
| Diaphragm | m. | 0.28, 0.47 (2) | | |
| | f. | 0.74±0.24 (4) | | |
| Bone | m/f | 7.43±1.16 (2m, 1f) | | |
| Muscle | m/f | 40.59±2.39 (2m, 1f) | | |

[a]Carcass = residual muscle and bone. m = male, f = female. ND = not done.

*Modification of adrenal metabolism* Attempts were made to influence tumour colonisation in the adrenals by blocking steroid production using aminoglutethimide in a 3-day schedule giving 250, 125, 62 $mg\,kg^{-1}$ on days −1 through to

**Table XIII** Comparison of % distribution of cardiac output with the % distribution of tumour cells 5 min after injection into anaesthetized rats

| | | *% Distribution cardiac output* | *% Distribution sarcoma cells* |
|---|---|---|---|
| Number of rats | m. | 8 | 5 |
| | f. | 7 | 6 |
| Carcass[a] | m. | 52.65 ± 12.73 | 38.33 ± 13.25 |
| | f. | 61.55 ± 4.35 | 38.46 ± 7.60 |
| Kidneys | m. | 9.81 ± 4.25 | 9.38 ± 4.30 |
| | f. | 6.84 ± 1.03 | 7.87 ± 1.06 |
| Heart | m. | 3.74 ± 2.61 | 6.13 ± 3.59 |
| | f. | 4.58 ± 2.00 | 7.22 ± 5.11 |
| Skin | m. | 4.56 ± 1.75 | 3.93 ± 1.48 |
| | f. | 3.18 ± 0.72 | 3.85 ± 1.10 |
| Brown fat | m. | 1.54 ± 1.46 | 0.72 ± 0.23 |
| | f. | 1.00 ± 0.47 | 0.54 ± 0.18 |
| Brain | m. | 3.39 ± 0.79 | 2.38 ± 1.13 |
| | f. | 3.75 ± 1.04 | 2.14 ± 0.60 |
| Adrenals | m. | 0.39 ± 0.11 | 0.39 ± 0.21 |
| | f. | 0.51 ± 0.16 | 0.33 ± 0.06 |
| Testes | m. | 1.14 ± 0.26 | 0.76 ± 0.12 |
| Ovaries | f. | 0.37 ± 0.29 | 0.34 ± 0.10 |
| Lungs | m. | 4.05 ± 1.72 | 18.46 ± 3.84 |
| | f. | 3.02 ± 1.82 | 16.83 ± 3.23 |
| Pancreas | m. | 1.59 ± 0.52 | 1.59 ± 0.64 |
| | f. | 1.03 ± 0.32 | 1.72 ± 0.45 |
| Spleen | m. | 0.94 ± 0.41 | 1.02 ± 0.45 |
| | f. | 0.95 ± 0.55 | 1.08 ± 0.44 |
| Small bowel | m. | 8.30 ± 3.58 | 4.78 ± 2.61 |
| | f. | 6.65 ± 1.39 | 5.48 ± 1.24 |
| Large bowel | m. | 2.59 ± 0.95 | 1.89 ± 1.40 |
| | f. | 1.94 ± 0.64 | 3.11 ± 1.12 |
| Stomach | m. | 1.28 ± 0.43 | 0.93 ± 0.30 |
| | f. | 0.73 ± 0.28 | 0.72 ± 0.25 |
| Mesentery | m. | 0.58 ± 0.31 | 0.35 ± 0.15 |
| | f. | 0.54 ± 0.15 | 0.46 ± 0.24 |
| Liver | m. | 2.65 ± 0.98 | 8.49 ± 3.27 |
| | f. | 2.30 ± 0.58 | 9.03 ± 3.57 |
| *Organs not measured in all animals. ( ) No. of rats* | | | |
| Diaphragm | m. | ND | 0.75 ± 0.10 (3) |
| | f. | ND | 0.59 ± 0.18 (6) |
| Jaw | m. | 0.04, 0.04 (2) | 0.03 ± 0.01 (3) |
| | f. | ND | 0.07 ± 0.05 (6) |

[a]Carcass = residual muscle and bone; m = male, f = female; ND = not done.

**Table XIV** Bioassays of organs for the presence of viable sarcoma MC28 cells in animals injected at different times before bioassay and comparison with the later development of tumour colonisation studied histologically

| | *Time following tumour cell injection (1 × 10^6 cells) at which the donor was killed and the organs transplanted i.p. into untreated recipients* | | | *Histological examination for tumour growth at 15 days* |
|---|---|---|---|---|
| | *5 min* | *1 day* | *5–6 days* | |
| Adrenals | 2/2[b] | 2/2 | 4/4 | Overt tumour |
| Kidneys | 2/2 | 4/4 | 4/4 | Microscopic tumour |
| Spleen | 6/6 | 2/2 | 0/4 | No tumour |
| Small int. | 1/2 | 1/2 | 0/3 | No tumour |
| Blood 1/2 ml. | 0/3 | – | 0/1 | No tumour |
| Liver | 3/4 | 0/2 | 0/4 | No tumour |
| Liver[a] | – | – | 0/5 | Tumour rarely |

[a]In these animals, $10^5$ cells had been injected i.ptl and not by the left ventricular route. [b]Incidence of i.p. tumours in recipients.

**Table XV** Distribution of human tumours following left ventricular injection into immunosuppressed female rats

| | *Human melanoma (HX 34)* | *Human melanoma (HX 46)* | *Human carcinoma (HX 70)* |
|---|---|---|---|
| No. of rats developing tumour colonies | 5/5 | 2/4 | 1/2 |
| Mode of immuno-suppression | Cyclosporin A | Nude rats | Cyclosporin A |
| No. of cells injected | $10^6$ | $10^6$ | $5 \times 10^6$ |
| Adrenals | 5/5 | 1/4 | 1/2 |
| Bone | 1/5 | 1/4 | 1/2 |
| Kidneys | 3/5 | 0/4 | 0/2 |
| Periodontal ligament | 0/5 | 1/4 | 0/2 |

No other tumour colonies found.

day +1 respectively; or 125 mg kg$^{-1}$ day$^{-1}$ from day 0 and metyrapone (at 125 mg kg$^{-1}$ day$^{-1}$ from day 0) but neither inhibited nor delayed the development of tumour growth in the adrenals or other sites (data not shown). Adrenergic beta blockade by propranol (2.5 mg kg$^{-1}$ day$^{-1}$ from day −1 to day +1) also did not alter the pattern or incidence of tumour growth (data not shown).

*Preferential growth at sites of trauma and healing*

In animals undergoing laparotomy followed by the i.a. inoculation of sarcoma MC28 it was found that tumour colonies localised to the muscle of the laparotomy wound (Table XIX). Traumatisation of the muscle (ie the laparotomy incision) when carried out 2 to 8 days prior to inoculation of tumour cells was most effective in promoting tumour growth while an incision at the time of cell injection was ineffective. A similar phenomenon was observed in 8/12 rats after the i.a. injection of $10^6$ breast carcinoma cells.

In 10 rats that had undergone laparotomy 4 days previously all developed tumour colonies in the muscle scar (an average of 4.5 nodules per scar) after the delivery of $10^5$ sarcoma MC28 cells but only 2 developed single tumour colonies in the rest of the skeletal muscle which received ~30% of delivered cells (see Table XII). Although the percentage of cardiac output going to muscle wounds was higher in the healing muscle −0.77 ± 0.17% as compared to 0.35 ± 0.19% in the same weight of adjacent non-traumatised muscle ($n = 5$ rats), this cannot account for the very marked localisation of tumour in the wounds. When quantified (as already done above for different organs) the probability of an arrested sarcoma cell in the wound developing into a colony is ~1 in 130 whereas in non-traumatised skeletal muscle it is ~1,000 times less likely.

Deliberate manipulation of the liver, partial hepatectomy or treatment with carbon tetrachloride before or around the time of arterial inoculation of sarcoma MC28 promoted liver tumour colony formation (Table XX). Manipulation at the time of i.ptl. injection of sarcoma MC28 similarly potentiated the incidence of development of tumour growths (Table XX).

*Promotion of tumour growth in the peritoneal cavity by pristane*

Since trauma potentiated bloodborne tumour colonisation and since this could not be explained by an increased delivery of cells alternative mechanisms were examined. The timing of the promotional effect of trauma in the case for muscle coincides with the time when macrophages infiltrate the wound and the importance of these was tested using an i.p. system. The peritoneal cavity is a favoured site for the

**Table XVI** Relative percentage distribution of cell associated radioactivity at different time points following left ventricular injection of labelled sarcoma MC 28 into conscious rats (Alcohol extracted)

| *Time after inject.* | *5 min* | *1 h* | *3 h* | *5 h* | *16 h* |
|---|---|---|---|---|---|
| *Total resid. counts* | *83.63%* | *68.66%* | *65.45%* | *66.57%* | *19.58%* |
| *No. rats* | *(n=3)* | *(n=4)* | *(n=3)* | *(n=3)* | *(n=4)* |
| Heart | 16.69±2.37 | 13.53± 2.33 | 12.70±1.61 | 19.59±7.17 | 15.36±11.77 |
| Kidney | 19.49±3.46 | 18.31± 4.51 | 28.67±6.60 | 26.70±5.11 | 50.06±11.06 |
| Brown fat | 11.61±2.37 | 10.48± 6.62 | 14.17±3.40 | 11.29±3.53 | 3.85± 1.05 |
| Brain | 4.61±2.09 | 5.68± 4.66 | 3.18±1.18 | 3.97±1.49 | 2.94± 1.49 |
| Adrenals | 0.41±0.05 | 0.38± 0.26 | 0.15±0.13 | 0.55±0.22 | 0.51± 0.29 |
| Lungs | 22.04±3.36 | 22.98±10.16 | 19.31±2.46 | 18.18±7.71 | 3.58± 0.39 |
| Spleen | 0.81±0.32 | 1.40± 0.20 | 1.07±0.28 | 1.24±0.27 | 1.14± 0.39 |
| Small bowel | 3.94±1.40 | 4.59± 1.87 | 4.79±1.07 | 3.99±0.91 | 6.7 ± 0.48 |
| Mesentery | 0.97±0.13 | 0.83± 0.05 | 1.19±0.44 | 1.78±1.36 | 0.37± 0.13 |
| Stomach | 2.03±0.62 | 2.61± 1.62 | 2.33±0.93 | 2.31±0.87 | 4.31± 1.54 |
| Large bowel | 3.38±0.72 | 3.13± 0.98 | 2.04±0.14 | 1.25±0.25 | 1.07± 0.24 |
| Liver | 14.02±1.25 | 16.08± 8.71 | 10.40±0.90 | 9.15±1.61 | 9.55± 1.60 |

Carcass, skin, pancreas, bladder, thymus and thyroid all excluded for technical reasons. The rest of the organs were totalled and called 100% at each time point.

**Table XVII** Relative vascularity in female rats

| | *Anaesthetized (n=7) Weight=185±34 g* | *Conscious (n=9) Weight=185±35 g* |
|---|---|---|
| *Organs with venous drainage to the lungs* | | |
| Adrenals | 14.85±5.30 | 8.61±2.86 |
| Heart | 13.95±6.87 | 15.49±5.05 |
| Ovaries | 10.38±8.96 | 5.07±3.04 |
| Left kidney | 8.34±1.38 | 14.12±4.44 |
| Right kidney | 8.33±1.22 | 12.73±5.11 |
| Left brain | 5.86±2.22 | 2.36±1.37 |
| Right brain | 2.88±0.90 | 2.82±1.24 |
| Hind brain | 4.27±1.77 | 2.21±0.78 |
| Thymus | 3.75±1.70 | 2.67±1.84 |
| Thoracic brown fat | 2.06±0.99 | 14.95±5.12 |
| Abdominal brown fat | 0.98±0.50 | 13.79±8.12 |
| Interscapular brown fat | 0.68±0.39 | 12.70±7.93 |
| Uterus | 1.81±1.95 | 0.74±0.49 |
| Carcass | 0.92±0.06 | 0.65±0.11 |
| Skin | 0.18±0.04 | 0.30±0.28 |
| Bladder | 0.07 | 0.22±0.27 |
| Lung | 5.09±3.43 | 1.43±1.18 |
| *Organs with venous drainage to the liver* | | |
| Spleen | 3.85±2.47 | 2.53±1.09 |
| Small bowel | 3.60±0.66 | 5.69±1.72 |
| Pancreas | 2.43±0.79 | 5.30±1.98 |
| Large bowel | 1.56±0.54 | 3.27±0.98 |
| Stomach | 0.91±0.22 | 2.62±1.15 |
| Small bowel mesentery | 0.58±0.20 | 0.93±0.51 |
| Liver | 0.46±0.11 | 0.12±0.10 |
| Liver including portal vein fraction | 2.80±0.32 | 3.63±1.12 |

growth of transplanted sarcomas and hepatomas which develop as solid tumours. Thus the $TD_{50}$ (the cell number to induce tumours in 50% of animals) for MC28 is $3 \times 10^4$ cells when inoculated i.m., of the order of $10^2$ when inoculated i.p. Moreover bloodborne tumour cells which are dormant in organs such as lung (Alexander *et al.*, 1985) will grow when transplanted into the peritoneal cavity.

Intraperitoneal tumour growth of the inoculated sarcomas and hepatoma was initially confined to the omentum as discrete nodules, before spread by direct extension to the mesentery, testicular or fallopian fat, the diaphragm, organ capsules and muscle wall. Pristane treatment of the rat's peritoneum produced a macrophage rich ascites which was maximal between 12 and 18 days later when the number of mononuclear cells from a peritoneal wash rose from $7.5 \times 10^6$ to $19 \times 10^7$. By 40 days the number of peritoneal leukocytes had returned to normal but the omentum was still enlarged and highly cellular. By 100 days the omentum had a normal appearance and MC28 sarcoma grew as in control rats.

Pre-treatment with pristance prior to i.p. injection of cells greatly facilitated tumour growth as measured by a reduction in $TD_{50}$, but was most dramatic in terms of tumour mass (Table XXI). Although this was difficult to quantify in the pristane treated groups as the tumour spread rapidly, by killing pristane treated rats at 7 and 9 days after tumour inoculation it could be seen that these tumours had their origin in the greater omentum.

**Table XVIII** Potentiating effect of dexamethazone on liver and kidney tumour colonisation following left ventricular injection of sarcoma MC 28

| Group | A | B | C | E | F | G | H |
|---|---|---|---|---|---|---|---|
| Day dex. started | −8 | | −1 | −4 to 0 | | +1 | +2 to +6 |
| Day dex. stopped | −1 | | 0 to +1 | +8 to +16 | | +16 | +16 |
| Kidney | 0/5 | 0/5 | 1/6 | 8/10 | 3/3 | 2/2 | 5/5 |
| Liver | 0/5 | 0/5 | 0/6 | 7/10 | 1/3 | 1/2 | 0/5 |
| Adrenal | 5/5 | 5/5 | 6/6 | 7/10 | 3/3 | 2/2 | 5/5 |
| Ovary | 5/5 | 5/5 | ND | ND | ND | ND | ND |
| Jaw | 3/5 | 1/5 | 3/6 | 4/10 | 2/3 | 1/2 | 2/5 |
| Bone | 2/5 | 4/5 | 4/6 | 4/10 | 2/3 | 1/2 | 5/5 |

The groups are divided according to the timing of the treatment in relation to the timing of the cell injection (day 0). Tumour colonies were occasionally found in the brown fat, lung, heart, muscle, skin, diaphragm and mesentery. Group A – $10^6$ cells injected. All other groups received $10^5$ cells. Group F – 0.1 mg $day^{-1}$ injections. All other groups were given 1 mg $day^{-1}$.

**Table XIX** Incidence of tumour colony formation in muscle wounds after left ventricular injection of $10^6$ sarcoma MC 28 cells

| *Timing of trauma in relation to tumour cell injection* | *Number of rats with tumour colonies in the muscle wound* |
|---|---|
| 8 days before | 7/8 |
| 5 days before | 2/2 |
| 4 days before | 6/6 |
| 2 days before | 0/2 |
| 1 day before | 1/8 |
| 5 min before | 1/6 |
| 5 min after | 1/12 |
| 1 day after | 1/6 |
| 2 days after | 0/8 |
| 3 days after | 1/4 |
| 4 days after | 0/2 |

30 rats had muscle laparotomies only but the remaining rats also had a variety of procedures including partial hepatectomy, liver manipulation and kidney manipulation.

Following i.c. injection of MC28 cells into normal rats we did not see tumour growth in the omentum but this was likely to be due to the very low delivery of cells to this organ (<0.05%). However when $1 \times 10^6$ cells were injected arterially into 5 rats treated with pristane 14 days previously 4 of these had omental tumour colonies

### Discussion

Our studies and those of others (eg Greene & Harvey 1964; Fidler, 1970; Proctor, 1976; Van de Velde *et al.*, 1977; Becker, 1978; Tarin & Price, 1979; Nanni *et al.*, 1983; Wilmott *et al.*, 1983; Barnet & Eccles, 1984) show that the lungs are with few exceptions the preferred site for bloodborne colonies from 'non-lymphoid' and 'non-haemopoietic' tumours injected into the systemic veins. Although the method in these studies whereby tumour cells are injected into vasculature is very artificial, the lung is also the commonest site for 'spontaneous' metastases derived from tumours implanted such that they develop systemic venous drainage (eg Ketcham *et al.*, 1961; Price *et al.*, 1982; Nanni *et al.*, 1983; Barnett & Eccles, 1984; Alterman *et al.*, 1985; Wingen & Schmahl, 1985). Following i.ptl. injection of tumour cells, the liver is likewise the preferred site of tumour growth – in both our studies and others (Fisher & Fisher, 1965; Vaage, 1973; Proctor, 1976; Tarin & Price, 1981; Dingemans & Roos, 1982).

The observation that colonisation of extrapulmonary and extrahepatic sites after i.v. or i.ptl. injection did not occur despite the ability of many organs to support tumour growth following the arterial delivery of low numbers of cells confirmed that the lung and liver capillary beds were trapping delivered tumour cells. This general pattern is also true for many other tumours injected via all three vascular routes, eg B16 melanoma (i.v. – Fidler, 1970; i.p. – Dingemans & Roos, 1982; i.a. – Weiss *et al.*, 1984), mouse mammary tumours (i.v. – Tarin & Price, 1979; i.p. – Tarin & Price, 1981; i.a. – Jaucaba *et al.*, 1983), mouse KHT sarcoma (i.v. – Siemann & Mulcahy, 1984; i.a. – Conley, 1979), mouse sarcoma (i.v., i.p. & i.a. – Vaage, 1973), rat allogeneic Walker carcinoma (i.v. – Agostino & Clifton, 1965; i.p. – Fisher & Fisher, 1965; i.a. – Sugarbaker, 1952), rat sarcoma, (i.v., i.p. & i.a. – Proctor, 1976).

Lymphoid or haemopoietic derived tumours behave differently and demonstrate the ability to traverse capillary beds since after i.v. injection they grow preferentially in sites distal to the lung such as the liver, spleen and bone (Potter *et al.*, 1957; Kobayashi *et al.*, 1962; Greene & Harvey, 1964; Pilgrim, 1969; Pilgrim, 1971; Parks, 1974; Sadler & Alexander, 1976; Brunson & Nicolson, 1978; Hart *et al.*, 1981; Willmott *et al.*, 1983; Konings *et al.*, 1985) and when labelled the radioactivity accumulates in the liver, spleen and intestines rather than the lungs (Hoelzer *et al.*, 1973; Sinha & Goldberg, 1974; Ioachim *et al.*, 1976; Sadler & Alexander, 1976). The cells of only a few 'solid' tumours demonstrate the ability to traverse the lung capillaries (some described by Greene & Harvey, 1963; Kinsey & Smith, 1959; Zeidman & Buss, 1952; Alessandri *et al.*, 1981) and also 'especially selected' variant cell lines derived from B16 murine melanoma (Fidler & Nicolson, 1976; Brunson *et al.*, 1978; Tao *et al.* 1979; Brunson & Nicolson, 1979; Raz & Hart, 1980; Fidler, 1984).

The mechanics of the circulation (Ewing's hypothesis) therefore determine the localisation of bloodborne colonies from 'solid tumours' in lung and liver after venous injection of cells and also determines the comparable localisation of metastases in the clinical situation (Murphy *et al.*, 1986, 1987). However, from our studies it is apparent that once tumour cells enter the arterial circulation it is 'soil' factors that determine the pattern of tumour growth (Paget's hypothesis). Weiss *et al.* (1984) observed similar results using the B16 melanoma in the mouse.

We have shown that arterially injected tumour cells distribute and arrest in parallel with the distribution of cardiac output. The results of our cardiac output studies are similar to those that have been published previously (eg Foster & Frydman, 1977; Tsuchiya *et al.*, 1978; Ishise *et al.*, 1980). However, the eventual pattern of tumour growth does not bear any relation to the pattern of tumour cell arrest or rate of autolysis. The likelihood of tumour cells surviving in different organs to become tumour growths (or 'soil effect') has been quantified in our studies and varies by a factor of several thousand between susceptible and refractory organs.

Various factors to explain site selectivity by bloodborne tumour have been postulated and include adhesion between tumour cells and their target organs (Netland & Zetter, 1984), biochemical factors (Nicolson & Dulski, 1986; Horak *et al.*, 1986), slow blood flow and attenuated endothelial linings (eg Tavassoli & Shaklai, 1979), the possession of different cell surfaces (Irimura & Nicolson, 1984), immunological factors (Hanna & Fidler, 1981), and the production in host tissues of growth factors needed for isolated cancer cells to grow (postulated by Alexander *et al.*, 1985).

Much of the work of Fidler (1984) and co-workers has emphasized the heterogenous nature of tumours and consequently the possibility that pre-existing variants in a tumour may have the potential to metastasize to specific sites. In contrast our studies and the results of the three

**Table XX** Effect of trauma on the development of sarcoma MC 28 liver tumour colonisation

| | *Timing of the trauma* | | | |
|---|---|---|---|---|
| | *15–1 day before cells* | *immediately before cells* | *Immediately after cells* | *1–7 days after cells* |
| *1. Tumour growth in rats injected with $10^6$ cells via the mesenteric veins*[a] | | | | |
| Manipulation | ND | 13/14 | 5/5 | 2/9 |
| Carbon tetrachloride | 3/3 | ND | ND | 0/3 |
| *2. Tumour growth in rats injected with $10^6$ cells via the left ventricle*[b] | | | | |
| Manipulation | 0/8 | 2/2 | 3/3 | 0/4 |
| Partial hepatectomy | 3/6 | 2/2 | 1/2 | 0/5 |
| Carbon tetrachloride | 2/2 | 1/1 | ND | ND |

[a]3/22 non-traumatised animals liver developed tumour colonisation; [b]1/57 non-traumatised animals developed liver tumour colonisation.

**Table XXI** Effect of stimulation of omentum by the mineral oil pristane on tumour growth in the peritoneal cavity

| *Tumour* | *No. of cells injected i.p.*[c] | *Time rat killed (days)*[a] | *Tumour size & incidence (no. with macroscopic tumours/no. in group)* Control | | |
|---|---|---|---|---|---|
| | | | *Control* | *Pristane treated*[b] | |
| MC28 sarcoma | 1,000 | 14 | 2/5** | 5/5*** | |
| | 500 | 21 | 2/5* | 5/5*** | |
| | 50 | 22 | 0/5 | 3/5*** | 2/5** |
| MC26 sarcoma | 5,000 | 31 | 2/5* | 5/5*** | |
| | 500 | 31 | 1/5* | 4/5** | |
| Hepatoma | 10,000 | 35 | 2/5* | 4/5*** | 1/5* |

*Tumour size*: *Between 1 to 10 small tumour nodules confined to the greater omentum; **Omentum extensively involved but no macroscopic tumour in other visceral organs; ***Tumour had spread to diaphragm, organ capsule and muscle wall. The peritoneum was widely involved which required the animals to be killed. [a]The time after cell inoculation when the animals were killed was in general the time when the first animals in the pristane treated group became noticeably unwell. This in most cases coincided with spread of tumour beyond the omentum; [b]Treatment with pristane: 2.5 ml i.p. for 200 g rats given between 12–17 days prior to i.p. inoculation of tumour cells; [c]To avoid growth of tumours along the needle track, the cells were injected in a relatively large (i.e. 1 ml) volume of fluid.

other studies in the rat in which tumour cells have been injected (Suemasu *et al.*, 1970; Sugarbaker, 1952; Proctor, 1976) show a broad similarity of arterial pattern of tumour colonisation. In man the 'arterial pattern' of carcinoma metastasis is also similar whatever the carcinoma type, whether it is primary lung tumour or lung metastasis discharging cells into the arterial circulation (Murphy *et al.*, 1987). Thus we have studied factors affecting organ environments rather than cell variants with specific site selectivities.

Our initial approach to the study of site selectivity was pharmacological. We observed that dexamethasone promoted sarcoma MC28 tumour colonisation in the liver and kidneys (organs which did not normally develop overt growth of tumour colonies) and hepatoma colonisation in the liver. The mechanisms of this effect remain unclear. If this promotion were secondary to the immunosuppressive effects of steroids, then it is not mediated by immunological factors relating to T-cell action since cyclosporin A did not have this promotional effect. Furthermore it does not seem to be mediated by the prostaglandin synthesis inhibiting action of steroids (Samuelsson *et al.*, 1979) since the non-steroidal anti-inflammatory flurbiprofen (also inhibiting prostaglandin synthesis – Heckford *et al.*, 1982) did not alter the arterial colonisation pattern. Hydrocortisone is known to facilitate the growth *in vitro* of freshly explanted cancer cells from human cancers and is routinely added to media used to clone human tumours. While it is tempting to attribute tumour colony promotion by dexamethasone in the kidney and liver to a mitogenic activity of corticosteroids, the failure of dexamethasone to induce tumour colonies at other sites such as intestines renders this hypothesis improbable.

Our second approach was to study the mechanisms underlying tumour growth in the adrenals. Preferential adrenal gland metastasis is not only a feature in the rat but is also observed in man (Murphy *et al.*, 1987), in the rabbit (Coman *et al.*, 1951; Knisely & Mahaley, 1958; Alexander & Altemeier, 1964), and in the mouse (Vaage, 1973; Conley, 1979; Jaucaba *et al.*, 1983; Weiss *et al.*, 1984). In the instances of extrapulmonary tumour growth occurring in mice after i.v. injection of tumour cells, adrenal or ovarian involvement is again fairly common (Fidler & Nicolson, 1976; Brunson *et al.*, 1978; Raz & Hart, 1980; Willmott *et al.*, 1983; Siemann & Mulcahy, 1984; Barnett & Eccles, 1984; Stackpole *et al.*, 1985; Alterman *et al.*, 1985). Our autoradiographic studies showed that the sarcoma MC28 tumour cells arrested in the periphery of the adrenal cortex – probably where the branching arterial subcapsular plexus breaks up into cortical capillaries (Coupland, 1974). Tumour growth was also usually in this region and this prompted the question as to whether the locally high concentration of steroids might be promoting tumour formation (especially since steroids potentiated growth in the liver and kidneys). Attempts to block steroid production in the adrenals with aminoglutethimide and metypyrone did not inhibit or delay the development of adrenal tumour colonies or colonies at other sites. However these drugs do not block steroid production completely (Temple & Liddle, 1970) and no definite conclusion is therefore possible. Adrenalectomy was also not found to delay the development of metastases at other sites suggesting that factors other than steroid concentration were more important in metastasis growth. We have not attempted to test the role of catecholamines by interfering with tissue levels but rather by blocking beta action with propranolol. However this did not alter the pattern or incidence of colony formation.

Trauma has been observed to facilitate the growth of bloodborne tumour and this confirms earlier studies of Robinson and Hoppe (1962), Alexander and Altemeier (1964) and Agostino and Clifton (1965). Although we have shown an increased delivery of blood (and hence tumour cells) to damaged and healing muscle in our experiments this is insufficient to explain the marked susceptibility of this muscle to bloodborne tumour growth. Tissue healing of all kinds can be broadly divided into 3 phases – an initial inflammatory (exudative) phase, a proliferative phase and a reorganisation or remodelling phase (Leibovich & Ross, 1975; Forrest, 1983). There is initial platelet activation and coagulation and then loss of fluid into the extravascular spaces. Neutrophils also extravasate and reach a peak level after 2 days and then decrease. Macrophages peak at about 3 days, persist for longer, and are important in both phagocytosis and stimulating tissue repair (Leibovich & Ross, 1975). This is then followed by a process of neovascularisation and repair or regeneration.

The timing of trauma potentiation of tumour colonisation in the muscle wounds coincided with the arrival of the macrophages and the possibility was considered that these, while promoting healing, might also promote tumour growth. Pristane introduced into the peritoneum will induce a general inflammatory reaction but it is characterised largely by a macrophage response. Pretreatment with pristane prior to i.p. tumour cell injection potentiated tumour growth. Macrophages might therefore be implicated in promoting tumour growth in these circumstances but the point is not proven. If this potentiation were secondary to the increased numbers of macrophages then it might be because of the many types of polypeptide growth factors they produce – including PDGF and EGF (Leslie *et al.*, 1984; Hamburger & White, 1986; Nordan & Potter, 1986; Morne *et al.*, 1986; Rich, 1986).

For trauma potentiation of liver tumour growth to occur tumour cell inoculation and trauma have to be timed much more closely, ie before the macrophage response has time to develop. This would indicate that promotion of growth of tumour emboli in the liver by trauma is unlikely to be caused by macrophages. Nor do we have any reason for attributing preference for tumour colonisation in normal non-traumatised organs such as adrenal or ovary to macrophages.

This investigation was supported by grants from the Cancer Research Campaign. We wish to acknowledge the help from Dr. S.A. Eccles of the Institute of Cancer Research who provided us with most of the tumours used.

### References

AGOSTINO, D. & CLIFFTON, E.E. (1965). Organ localization and the effect of trauma on the fate of circulating cancer cells. *Cancer Res.*, **25**, 1728.

ALESSANDRI, G., GIAVAZZI, R., FALAUTANO, P., SPREAFICO, F., GARATTINI, S. & MANTOVANI, A. (1981). A murine ovarian tumour with unique metastasizing capacity. *Eur. J. Cancer*, **17**, 651.

ALEXANDER, J.W. & ALTEMEIER, W.A. (1964). Susceptibility of injured tissues to hematogenous metastases. *Ann. Surg.*, **159**, 933.

ALEXANDER, P. (1976). The functions of the macrophage in malignant disease. *Ann. Rev. Med.*, **27**, 207.

ALEXANDER, P. (1983). Dormant metastases – studies in experimental animals. *J. Pathol.*, **141**, 379.

ALEXANDER, P., SENIOR, P.V., MURPHY, P. & CLARKE, R. (1985). Role of growth stimulatory factors in determining the sites of metastasis. In *Mechanisms of Cancer Metastasis*, Honn *et al.* (eds), Chapter 12. Kluwer Nijhoff: Boston.

ALTERMAN, A., FORNABAIO, D.M. & STACKPOLE, C.W. (1985). Metastatic dissemination of B16 melanoma: Pattern and sequence of metastasis. *J. Natl Cancer Inst.*, **75**, 691.

BARNETT, S.C. & ECCLES, S.A. (1984). Studies of mammary carcinoma metastasis in a mouse model system. I: Derivation and characterization of cells with different metastatic properties during tumour progression *in vivo*. *Clin. Exp. Metast.*, **2**, 15.

BECKER, F.F. (1978). Patterns of spontaneous metastasis of transplantable hepatocellular carcinomas. *Cancer Res.*, **38**, 163.

BRUNSON, K.W. & NICOLSON, G.L. (1978). Selection and biologic properties of malignant variants of a murine lymphosarcoma. *J. Natl Cancer Inst.*, **61**, 1499.

BRUNSON, K.W., BEATTIE, G. & NICOLSON, G.L. (1978). Selection and altered tumour cell properties of brain-colonising metastatic melanoma. *Nature*, **272**, 543.

BRUNSON, K.W. & NICOLSON, G.L. (1979). Selection of malignant melanoma variant cell lines for ovary colonization. *J. Supramol. Struct.*, **11**, 517.

COMAN, D.R., EISENBERG, R.B. & McCUTCHEON, M. (1949). Factors affecting the distribution of tumour metastases. Experiments with V2 carcinoma of rabbits. *Cancer Res.*, **9**, 649.

CONLEY, F.K. (1979). Development of a metastatic brain tumour model in mice. *Cancer Res.*, **39**, 1001.

COUPLAND, R.E. (1974). Blood supply of the adrenal gland. In *Handbook of Physiology – Endocrinology VI*, Chapter 20, p. 283. American Physiology Society. Williams and Wilkins: Baltimore.

DINGEMANS, K.P. & ROOS, E. (1982). Ultrastructural aspects of liver invasion (II). In *Liver Metastasis*, Weiss, L. & Gilbert, H.A. (eds) p. 51. G.K. Hall and Co.: Boston.

EWING, J. (1928). *Neoplastic diseases*, p. 86, 3rd edition. W.B. Saunders: London and Philadelphia.

FIDLER, I.J. (1970). Metastasis: Quantitative analysis of distribution and fate of tumour emboli labeled with $^{125}$I-5-Iodo-2′-deoxyuridine. *J. Natl Cancer Inst.*, **45**, 773.

FIDLER, I.J. & NICOLSON, G.L. (1976). Organ selectivity for implantation survival and growth of B16 variant tumor lines. *J. Natl Cancer Inst.*, **57**, 1199.

FIDLER, I.J. (1984). The evolution of biological heterogeneity in metastatic neoplasms. In *Cancer Invasion and Metastasis: Biologic and Therapeutic Aspects*, Nicholson, G. & Milas, L. (eds) p. 5. Raven Press: New York.

FISHER, E.R. & FISHER, B. (1965). Experimental study of factors influencing development of hepatic metastases from circulating tumor cells. *Acta Cytol.*, **9**, 146.

FORREST, L. (1983). Current concepts in soft connective tissue wound healing. *Br. J. Surgery*, **70**, 134.

FOSTER, D.O. & FRYDMAN, M.L. (1977). Comparison of microspheres and 86Rb as tracers of the distribution of cardiac output in rats indicates invalidity of 86Rb-based measurements. *Canad. J. Physiol. Pharmacol.*, **56**, 97.

GREENE, H.S.N. & HARVEY, E.K. (1964). The relationship between the dissemination of tumour cells and the distribution of metastases. *Cancer Res.*, **24**, 799.

HAMBURGER, A. & WHITE, C. (1986). Growth factors for human tumor clonogenic cells elaborated by macrophages isolated from human malignant effusions. *Cancer Immunol. Immunother.*, **22**, 404.

HANNA, N. & FIDLER, I.J. (1981). Relationship between metastatic potential and resistance to natural killer cell-mediated cytotoxicity in three murine tumor systems. *J. Natl Cancer Inst.*, **66**, 1183.

HART, I.R., TALMADGE, J.E. & FIDLER, I.J. (1981). Metastatic behaviour of a murine reticulum cell sarcoma exhibiting organ-specific growth. *Cancer Res.*, **41**, 1281.

HECKFORD, S.E., ECCLES, S.A., POWLES, T.J. & ALEXANDER, P.A. (1982). Failure of therapeutic effect of cyclophosphamide against rodent sarcomas and a leukaemia. *Br. J. Cancer*, **46**, 51.

HOELZER, D., CALVO, W., MEYER-HAMME, K.D. & HARRISS, E.B. (1973). Cell distribution and proliferation pattern of transferable acute leukaemia in rats. *J. Natl Cancer Inst.*, **50**, 1545.

HORAK, E., DARLING, D.L. & TARIN, D. (1986). Analysis of organ-specific effects on metastatic tumor formation by studies *in vitro*. *J Natl Cancer Inst.*, **76**, 913.

IOACHIM, H.L., PEARSE, A. & KELLER, S.E. (1976). Role of immune mechanisms in metastatic patterns of haemopoetic tumours in rats. *Cancer Res.*, **36**, 2854.

IRIMURA, T. & NICOLSON, G.L. (1984). Carbohydrate chain analysis by lectin binding to electrophoretically separated glycoproteins from murine B16 melanoma sublines of various metastatic properties. *Cancer Res.*, **44**, 791.

ISHISE, S., PEGRAM, B.L., YAMAMOTO, J., KITAMURA, Y. & FROHLICH, E.D. (1980). Reference sample microsphere method: Cardiac output and blood flows in conscious rat. *Am. J. Physiol.*, **239**, H443.

JUACABA, S.F., JONES, L.D. & TARIN, D. (1983). Organ preference in metastatic colony formation by spontaneous mammary carcinoma after intra-arterial inoculation. *Invasion Metast.*, **3**, 208.

KETCHAM, A.S., KINSEY, D.L., WEXLER, H. & MANTEL, N. (1961). The development of spontaneous metastases after removal of a 'primary' tumour. *Cancer*, **14**, 875.

KINSEY, D.L. & SMITH, R.R. (1959). Contrasts between human and experimental melanomas. *Surg. Gynaecol. Obstet.*, **109**, 539.

KNISELY, W.H. & MAHALEY, M.S. (1958). Relationship between size and distribution of 'spontaneous metastases' and three sizes of intravenously injected particles of VX2 carcinoma. *Cancer Res.*, **18**, 900.

KOBAYASHI, H., POTTER, M. & DUNN, T.B. (1962). Bone lesions produced by transplanted plasma-cell tumours in BALB/c mice. *J. Natl Cancer Inst.*, **28**, 649.

KONINGS, A.W.T., DE JONG, B. & HULSTAERT, C.E. (1985). Different homing patterns of isolated lymphoma cells: Relationship with surface morphology and chromosomal aberrations. In *Treatment of metastasis: Problems and Prospects*, Hellmann, K. & Eccles, S.A. (eds). Taylor and Francis: London and Philadelphia.

LEAK, L.V., POTTER, M. & MAYFIELD, W.J. (1985). Response of the peritoneal mesothelium to the mineral oil, pristane. In *Current Topics in Microbiology and Immunology*, Vol **22**. Springer Verlag, Berlin: Heidelberg.

LIEBERMANN-MEFFERT & WHITE, H. (1983). In: The Greater Omentum, publ. Springer.

LEIBOVICH, S.J. & ROSS, R. (1975). The role of the macrophage in wound repair. A study with hydrocortisone and antimacrophage serum. *Am. J. Pathol.*, **78**, 71.

MORNE, J. *et al.* (1986). Spontaneous expression of the c-*sis* gene and release of a platelet-derived growth factorlike molecule by human alveolar macrophages. *J. Clin. Invest.*, **78**, 61.

NORDAN, R. & POTTER, M. (1986). A macrophage-derived growth factor required by plasmacytomas for survival and proliferation *in vitro*. *Science*, **233**, 566.

MURPHY, P., ALEXANDER, P., KIRKHAM, N., FLEMING, J. & TAYLOR, I. (1986). Pattern of spread of bloodborne tumour. *Br. J. Surg.*, **73**, 829.

MURPHY, P., TAYLOR, I. & ALEXANDER, P. (1987). Pattern of bloodborne metastasis in man. *Hospital Update*, **13**, 686.

NANNI, P., DE GIOVANNI, C., LOLLINI, P., NICOLETI, G. & PRODI, G. (1983). TS/A: A new metastasizing cell line roma BALB/c spontaneous mammary adenocarcinoma. *Clin. Exp. Metast.*, **1**, 373.

NETLAND, P.A. & ZETTER, B.R. (1984). Organ-specific adhesion of metastatic tumor cells *in vitro*. *Science*, **224**, 1113.

NICOLSON, G.L. & DULSKI, K.M. (1986). Organ specificity of metastatic tumour colonization is related to organ-selective growth properties of malignant cells. *Int. J. Cancer*, **38**, 289.

PAGET, S. (1889). The distribution of secondary growth in cancers of the breast. *Lancet*, **i**, 571.

PARKS, R.C. (1974). Brief communication: Organ-specific metastasis of a transplantable reticulum cell sarcoma. *J. Natl Cancer Inst.*, **52**, 971.

PILGRIM, H.I. (1969). The kinetics of organ-specific metastasis of a transplantable reticulum cell sarcoma. *Cancer Res.*, **29**, 1200.

PILGRIM, H.I. (1971). The metastatic behaviour of a spleen-tropic reticulum cell sarcoma in splenectomized mice. *Proc. Soc. Exp. Biol. Med.*, **138**, 178.

POTTER, M., FAHEY, J.L. & PILGRIM, H.I. (1957). Abnormal serum protein and bone destruction in transmissible mouse plasma cell neoplasm. *Proc. Soc. Exp. Biol. Med.*, **94**, 327.

PRICE, J.E., CARR, D., JONES, C.D., MESSER, P. & TARIN, D. (1982). Experimental analysis of factors affecting spread using naturally occuring tumours. *Invasion Metast.*, **2**, 77.

PROCTOR, J.W. (1976). Rat sarcoma model supports both 'soil seed' and 'mechanical' theories of metastatic spread. *Br. J. Cancer*, **34**, 651.

RAZ, A. & HART, I.R. (1980). Murine melanoma: A model for intracranial metastasis. *Br. J. Cancer*, **42**, 331.

RICH, I. (1986). A role for the macrophage in normal haemopoesis. I. Functional capacity of bone-marrow-derived macrophages to release haemopoietic growth factors. *Exp. Hematol.*, **14**, 738.

ROBINSON, K.P. & HOPPE, E. (1962). The development of bloodborne metastases. Effect of local trauma and ischaemia. *Arch. Surg.*, **85**, 720.

ROGERS, A.W. (1969). In *Techniques of Autoradiography* (2nd edition). Elsevier Publishing: London and New York.

SADLER, T.E. & ALEXANDER, P. (1976). Trapping and destruction of blood-borne syngeneic leukaemia cells in lung, liver and spleen of normal and leukaemic rats. *Br. J. Cancer*, **33**, 512.

SAMUELSSON, B., HAMMERSTROM, S. & BORGEAT, P. (1979). Pathway of arachidonic acid metabolism. In: *Advances in Inflammation Research*, Vol **1**, Weissman, G. (ed) p.0. Raven Press: New York.

SENIOR, P.V., MURPHY, P. & ALEXANDER, P. (1985). Oestrogen dependent rat mammary carcinoma as a model for dormant metastases. In *Treatment of metastasis: Problems and Prospects*, Hellmann, K. & Eccles, S.A. (eds) p. 113. Taylor and Francis: London and Philadelphia.

SIEMANN, D.W. & MULCAHY, R.T. (1984). Characterization of growth and radiation response of KHT tumor cells metastatic from lung to ovary and kidney. *Clin. Exp. Metastasis*, **2**, 73.

SINHA, B.K. & GOLDENBERG, G.J. (1974). The effect of trypsin and neuraminidase on the circulation and organ distribution of tumour cells. *Cancer*, **34**, 1956.

STACKPOLE, C.W., ALTERMAN, A.L. & FORNABAIO, D.M. (1985). Growth characteristics of clonal cell populations constituting a B16 melanoma metastasis model system. *Invasion. Metast.*, **5**, 125.

SUEMASU, K., KATAGIRI, M., SHIMOSATO, Y., MIKUNI, M. & ISHIKAWA, S. (1970). Initial stage of haematogenous metastasis of $^{51}$Cr-labelled tumor cells. *Gann.*, **61**, 7.

SUGARBAKER, E.D. (1952). The organ selectivity of experimentally induced metastases in rats. *Cancer*, **5**, 606.

TAO, T., MATTER, A., VOGEL, K. & BURGER, M. (1979). Liver colonizing cells selected from B16 melanoma. *Int. J. Cancer*, **23**, 854.

TARIN, D. & PRICE, J.E. (1979). Metastatic colonization potential of primary tumour cells in mice. *Br. J. Cancer*, **39**, 740.

TARIN, D. & PRICE, J.E. (1981). Influence of the microenvironment and vascular anatomy on 'metastatic' colonization potential of mammary tumors. *Cancer Res.*, **41**, 3604.

TAVASSOLI, M. & SHAKLAI, M. (1979). Absence of tight junctions in endothelium of marrow sinuses: Possible significance for marrow cell egress. *Br. J. Haematol.*, **41**, 303.

TEMPLE, E.T. & LIDDLE, G.W. (1970). Inhibitors of adrenal steroid biosynthesis. *Ann. Rev. Pharmacol.*, **10**, 199.

TSUCHIYA, M., FERRONE, R.A., WALSH, G.M. & FROHLICH, E.D. (1978). Regional blood flows measured in conscious rats by combined Fick and microsphere methods. *Am J. Physiol.*, **235**, H357.

VAAGE, J. (1973). Humoral and cellular immune factors in the systemic control of artificially induced metastases in C3HF mice. *Cancer Res.*, **33**, 1957.

VAN DE VELDE, C.J.H., VAN PUTTEN, L.M. & ZWAVELING, A. (1977). A new metastasizing mammary carcinoma model in mice. Model characteristics and applications. *Eur. J. Cancer*, **13**, 555.

VIADANA, E., BROSS, I.D.J. & PICKREN, J.W. (1978). Cascade spread of blood-borne metastases in solid and non-solid cancers of humans. In *Pulmonary Metastasis*, Weiss, L. & Gilbert, H.A. (eds) p. 142. G.K. Hall: Boston, Massachusetts.

WEISS, L., BRONK, J., PICKREN, J.W. & LANE, W.W. (1981). Metastatic patterns and target organ arterial blood flow. *Invasion Metast.*, **1**, 126.

WEISS, L., WARD, P.M., HARLOS, J.P. & HOLMES, J.C. (1984). Target organ patterns of tumours in mice following the arterial dissemination of B16 melanomas. *Int. J. Cancer*, **33**, 825.

WILLMOTT, N., MALCOLM, A., McLEOD, T., GRACIE, A. & CALMAN, K.C. (1983). Changes in anatomical distribution of tumour lesions induced by platelet-active drugs. *Invasion Metast.*, **3**, 32.

WINGEN, F. & SCHMAHL, D. (1985). A transplantable osteosarcoma in rats as a model for chemotherapy. In *Treatment of Metastasis: Problems and Prospects*, Hellman, K. & Eccles, S.A. (eds). Taylor and Francis: London and Philadelphia.

ZEIDMAN, I. & BUSS, J.M. (1952). Transpulmonary passage of tumor cell emboli. *Cancer Res.*, **12**, 731.